# Central venous pressure estimation from ultrasound assessment of the jugular venous pulse

**DOI:** 10.1371/journal.pone.0240057

**Published:** 2020-10-28

**Authors:** Paolo Zamboni, Anna Maria Malagoni, Erica Menegatti, Riccardo Ragazzi, Valentina Tavoni, Mirko Tessari, Clive B. Beggs

**Affiliations:** 1 HUB Center Regione Emilia Romagna for Venous and Lymphatics Disorders, University Hospital of Ferrara, Ferrara, Italy; 2 Department of Morphology, Surgery and Experimental Medicine, University of Ferrara, Ferrara, Italy; 3 Institute for Sport, Physical Activity and Leisure, Leeds Beckett University, Leeds, United Kingdom; Temple University, UNITED STATES

## Abstract

**Objectives:**

Acquiring central venous pressure (CVP), an important clinical parameter, requires an invasive procedure, which poses risk to patients. The aim of the study was to develop a non-invasive methodology for determining mean-CVP from ultrasound assessment of the jugular venous pulse.

**Methods:**

In thirty-four adult patients (age = 60 ± 12 years; 10 males), CVP was measured using a central venous catheter, with internal jugular vein (IJV) cross-sectional area (CSA) variation along the cardiac beat acquired using ultrasound. The resultant CVP and IJV-CSA signals were synchronized with electrocardiogram (ECG) signals acquired from the patients. Autocorrelation signals were derived from the IJV-CSA signals using algorithms in R (open-source statistical software). The correlation r-values for successive lag intervals were extracted and used to build a linear regression model in which mean-CVP was the response variable and the lagging autocorrelation r-values and mean IJV-CSA, were the predictor variables. The optimum model was identified using the minimum AIC value and validated using 10-fold cross-validation.

**Results:**

While the CVP and IJV-CSA signals were poorly correlated (mean r = -0.018, SD = 0.357) due to the IJV-CSA signal lagging behind the CVP signal, their autocorrelation counterparts were highly positively correlated (mean r = 0.725, SD = 0.215). Using the lagging autocorrelation r-values as predictors, mean-CVP was predicted with reasonable accuracy (r^2^ = 0.612), with a mean-absolute-error of 1.455 cmH_2_O, which rose to 2.436 cmH_2_O when cross-validation was performed.

**Conclusions:**

Mean-CVP can be estimated non-invasively by using the lagged autocorrelation r-values of the IJV-CSA signal. This new methodology may have considerable potential as a clinical monitoring and diagnostic tool.

## Introduction

The jugular venous pulse (JVP), a pivotal parameter of efficient cardiac function, reflects pressure variation in the right atrium over the cardiac cycle [1]. It is represented by changes in the cross-sectional area (CSA) of the internal jugular veins (IJVs) that can be easily monitored using high resolution B-mode sonography [2, 3]. The IJVs readily respond to changes in transmural pressure because they are thin-walled floppy vessels, with the result that the CSA of these vessels fluctuates in a cyclical motion that is influenced by both the cardiac and respiratory cycles [2]. However, despite its importance, JVP evaluation is often neglected in clinical practice [4], with central venous pressure (CVP) (i.e. the pressure in the right atrium and ventricle at the end of diastole) being the parameter most commonly evaluated. CVP is a variable indicative of cardiovascular function, having the dual role of distending the diastolic right ventricle and opposing venous return [5]. As such, CVP is a useful guide for assessing cardiac preload and vascular volume status, as well as being an indicator that can assist in better understanding the reasons for changes in cardiac output, given the interaction that exists between cardiac function and venous return [6]. Its measurement remains widely used in intensive care units and emergency centers mainly for guiding fluid administration in patients with haemodynamic instability [7]. CVP is invasively acquired, requiring the insertion of a catheter via the IJV or subclavian veins. Furthermore, the procedure has inherent drawbacks because it requires high levels of skill and carries a significant risk of complication [8]. Consequently, there is a need to develop a non-invasive methodology for accurately assessing CVP.

Significant correlations have been reported between CVP and: (i) IJV-CSA [9, 10]; (ii) the IJV/common carotid artery CSA ratio [9–11]; and (iii) the external jugular venous pulse [12]. This led us to hypothesize that it should be possible to predict CVP from the JVP [13]. Indeed, several methods have been reported for monitoring CVP non-invasively [12, 14–18], although none, with the exception of near-infrared spectroscopy (NIRS) [12], have demonstrated sufficient accuracy and precision or ease of use. Recently an ultrasonographic (US) technique for obtaining the JVP from a high-resolution B-mode sonogram sequences (US-JVP), recording the changes in IJV-CSA over the cardiac cycle (CC), has been proposed [19], which appears to have potential as an approach for estimating CVP. We therefore undertook the exploratory study presented here with the aim of testing the efficacy (proof-of-concept) of a new methodology (also presented here) for estimating CVP purely from changes in IJV-CSA acquired through US investigation. This methodology, which utilizes the autocorrelation signal of the JVP (i.e. changes in IJV-CSA over the CC) and reflects the spectral characteristics of the CVP pulse, is described in the *Theory* part of the *Methods* section below, which outlines the theory underpinning the autocorrelation technique. Importantly, the methodology is generic and can be applied to individual patients, without any prior knowledge of the CVP pulse or of the patient’s anatomical features (e.g. length of neck, etc).

## Methods

The study was performed prospectively by a team composed of clinicians, technicians, physicists and bio-engineers, and included a data collection phase, a post-processing phase and a data analysis phase. The study was part of a project granted by the Italian Health Ministry (Ricerca Finalizzata 2013, RF-2013-02358029) that was approved by the Ethics Committee of Ferrara, Italy (reference No.160499). The study was carried out in accordance with the ethical guidelines on good clinical practice as laid down in European Directive and the Declaration of Helsinki. The trial was registered NCT03917368 (https://clinicaltrials.gov/ct2/show/NCT03917368).

### Data collection

The data collection phase, implemented at the University Hospital of Ferrara (Italy), included the direct invasive measurement of the CVP and the US evaluation of the JVP. Both assessments were performed at the same time.

### Subjects

From September 2016 to July 2018, fifty-eight consecutive subjects (21 males and 37 females; mean age 61 ± 3 years) were enrolled as the sample study group. Participants were selected from hospitalised adult patients at University Hospital of Ferrara (Italy) requiring a scheduled central venous catheterisation (CVC) and measurement of the CVP as part of their usual care. Inclusion criteria were: age ≥18 years, spontaneous breathing and capacity to give informed consent. Patients were excluded if they needed a cannulation via the internal jugular venous access in order not to interfere with the ultrasound assessment, or if they were pregnant. In addition, because high quality US images and JVP trace data were required in order to develop the diagnostic model, any subjects who yielded corrupted or technically imperfect images or signals were also excluded. All participants were approached before the CVC procedure and signed a written informed consent form.

### Direct invasive measurement of the CVP

In a surgery room, after moderate sedation (midazolam 4 mg intravenous) and subcutaneous infiltration of local anaesthetics (mepivacaine 1% 20 ml), patients undertook a standardized subclavian tunnelled catheterization by infraclavicular approach, under fluoroscopic control. Following the catheterisation, patients were placed out of the surgery room lying completely supine on a bed in order to measure the CVP according to the standard [20]. The central venous catheter was connected to intravenous fluid within a pressure bag inflated up to 300 mmHg. The pressure measurement system was placed on the right or left arm at the level of the fifth rib (phlebostatic axis) and the zero reference was checked and calibrated when necessary. Three electrodes were placed on patients’ chests for the simultaneous assessment of the electrocardiogram (ECG) signal. The pressure line and the ECG cables were connected to a standard analogue monitor (Philips M3046A M4, Philips Medical System, Boeblingen, Germany), which in turn was connected to a video grabber system, allowing the capture and storage of the screen images on a computer, as they were not directly accessible in their digital format. A single investigator performed all the central venous catheter measurements, which served as the ‘gold standard’ measurement of CVP value and waveform.

### US assessment of the IJVs

Alongside the direct CVP measurement, the US assessment of the IJVs (both right and left) was performed as previously described [3, 21]. A Vivid-q ultrasound system (GE Medical Systems, Horten, Norway) equipped with a linear array probe L12-RS (7.5–11 MHz) was used. Patients were asked to maintain their neck in a fixed position on the longitudinal axis, avoiding flexion, hyperextension and rotation, which might compress the veins and influence the measurements. The probe, smeared with a copious amount of gel, was placed on the patients’ neck in a transverse plane with respect to the length of the vessels, at the level of C5-C6, the so-called J2 plane [22]. The IJV-CSA was insonated trying to avoid any pressure on the vessel and a B-mode video-clip of 10–15 sec was recorded and stored. This was sufficient time to record several cardiac cycles and two or three respiratory cycles. The ECG trace was automatically recorded contextually with the IJV-CSA B-mode images. All the US evaluations were performed by a single investigator.

### Post-processing

**Fig 1:**
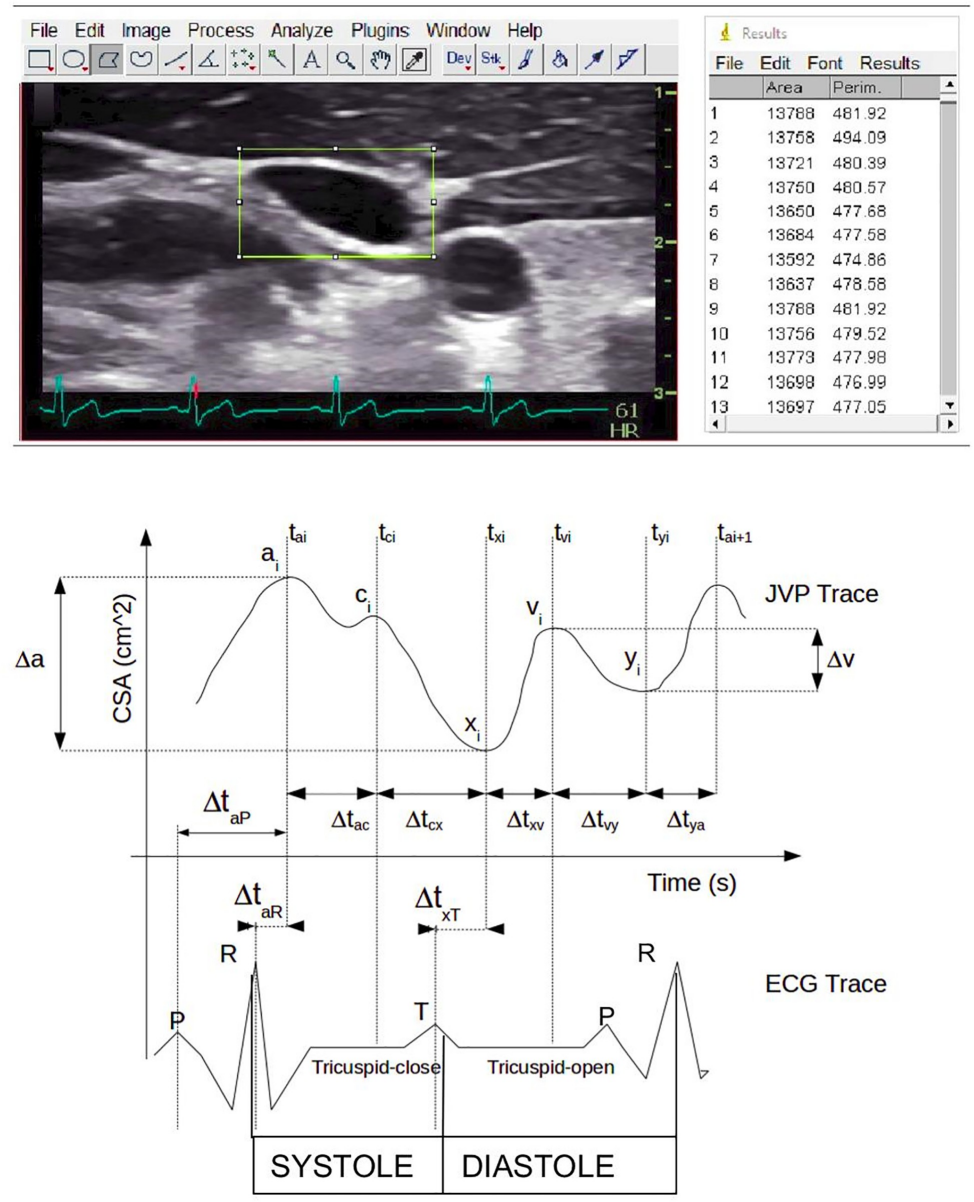
ImageJ custom plugin to detect area variations: The top figure shows an example of B-mode image with a rectangular Region of Interest (ROI) and synchronized ECG trace, together with a list of ROIs and collected measurements. The bottom figure shows the indicative JVP trace with a, c, v peaks and x, y troughs synchronized with the ECG trace. Systole and diastole phase as well as the corresponding opening and closure of the tricuspid valve and various time intervals (Δt) are also shown.

The stored US IJV video clips and the CVP images were processed off-line to obtain a numerical dataset to enable the subsequent analyses and formulation of models. First of all, images and video clips were elaborated with the software *ImageJ* [23] (e.g. Fig 1). The procedure to obtain an IJV-CSA dataset of signals over time consisted of several passages both manual and automatic. This procedure provided the IJV-CSA values in cm^2^ versus sonogram acquisition time, the obtained result corresponded to the JVP trace (e.g. Fig 1) [3, 19, 21], whereas the CVP time series signal dataset was produced by digitally identifying the position of each point of the CVP trace represented on the acquired image. Subsequently the JVP and the CVP traces were synchronised using the ECG signal recorded together with the measurements. Finally, the obtained datasets were elaborated with Matlab (MathWorks, Inc.) and R software [24] to remove the frequencies of non-cardiac origin, mainly CSA variations due to activation of the thoracic pump [25]. This removed noise from the traces, therefore highlighting the contribution of the cardiac contraction to both traces [21, 26]. Finally, the post processing aimed to conform the length of the traces making the comparison easier.

### Theory

While the relationship between the IJV-CSA and CVP signals is difficult to interpret in the time domain, it has been shown that in the frequency domain the two signals share similar spectral characteristics [27]. Therefore, in theory it should be possible to determine a great deal about the CVP signal purely through examination of the IJV pulse. However, factors such as respiration influence the IJV pulse and there is a phase lag between the CVP pulse and the IJV pulse, which makes it difficult to interpret the relationship between the two signals.

Autocorrelation, which assesses the degree of similarity between a given time series signal and a lagged version of itself over successive time intervals, can be used to gain insights into the spectral characteristics of signals. Through the Wiener–Khinchin theorem it can be shown that the Fourier transform (used in Fourier analysis) is closely related to the autocorrelation function of a signal [28]. As such, the autocorrelation signal can be used to assess the periodicity of any given time series signal using Eq 1.

$$f=\frac{1}{D\times dt}$$ (1)

Where: *f* is the frequency of the time series signal; *D* is the lag distance between successive peaks in the autocorrelogram; and *dt* is the sampling interval.

Because the autocorrelation function reflects the spectral characteristics of signals, if two time series signals such as the IJV and CVP pulses share similarities in the frequency domain, then their respective autocorrelation signals will exhibit similarities and will tend to be aligned irrespective of any lag between the two signals in the time domain. This suggests that the autocorrelation signal might be a useful tool with which to predict the characteristics of the CVP pulse using the IJV pulse.

For any given time series signal the corresponding autocorrelation signal is simply a plot of the changes in the correlation r-value as a function of delay. The autocorrelation r-value for any given lag in a time series can be computed using Eq 2.

$$r_{h}=\frac{\sum [\left(y_{t}-\bar{y})(y_{t+h}-\bar{y}\right)]}{\sum [\left({\left(y_{t}-\bar{y}\right)}^{2}\right)]}$$ (2)

Where: *r*_*h*_ is the r-value for any given lag; *y*_*t*_ is the time series signal at lag = 0; *y*_*t+h*_ is the time series signal at lag = h; and $\bar{y}$ is the mean of the time series signal.

From this it can be seen that the autocorrelation signal is strongly influenced by the mean of the measured signal in the time domain. Therefore, it can be hypothesized that it should be possible to estimate the mean of the CVP pulse directly from the autocorrelation signal of the IJV pulse, provided that the two share similar spectral characteristics.

### Data analysis

Signal processing and statistical analysis were undertaken using in-house algorithms written in R [24]. The relationship between the IJV-CSA and CVP time series signals for the respective subjects was assessed using Pearson correlation analysis, with the time lag between the two signals computed using the ‘ccf’ cross-correlation function in R. For each patient, the autocorrelation signals were derived from the IJV-CSA and CVP time series signals using the ‘acf’ function in R. From these, the correlation r-values for successive lags of 20 time intervals from 0 to 360 (i.e. 0, 20, 40, 60, … 360 intervals) were extracted and used to build the CVP prediction model. Fourier analysis was also performed using the ‘fft’ function in R to produce periodograms for each patient. From these the five most dominant frequencies (with amplitudes) were extracted for each patient. A linear regression model was then produced with mean CVP as the response (dependent) variable and the extracted autocorrelation r-values for the successive lags, together with the mean IJV-CSA value, as the predictor (independent) variables. The model was then refined using an in-house algorithm, which assessed all possible combinations of the predictor variables to identify the model with the minimum Akaike information criterion (AIC). The refined linear model was then used to predict the mean CVP for the respective subjects. Finally, in order to assess the general applicability of the refined model and to test its robustness as a predictor of mean CVP, 10-fold cross-validation was performed. This gave an indication of how well the model might perform in ‘real life’ clinical situations. In addition, a *post-hoc* statistical power calculation was performed using Cohen’s *f*^2^ with alpha = 0.05.

In order to test its clinical efficacy, the regression model was further evaluated by splitting the outcomes into three categories: (i) CVP within the normal range, 2–8 mmHg (2.72–10.88 cmH2O) [29, 30]; (ii) CVP above the normal range; and (iii) CVP below the normal range. For each class the sensitivity score was computed, together with overall accuracy achieved by the model, which was also statistically evaluated using Cohen’s Kappa statistic for measuring agreement.

## Results

**Fig 2:**
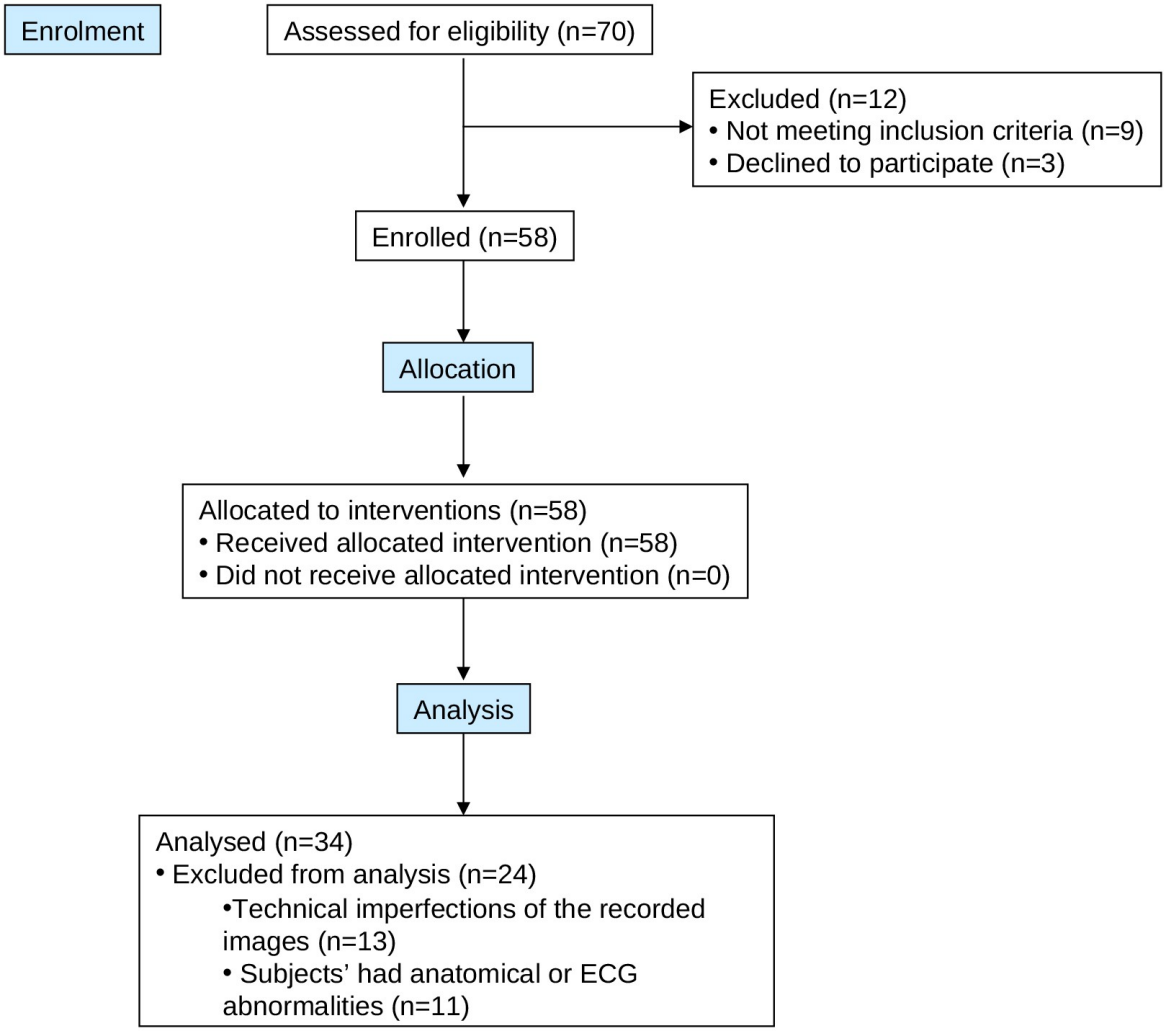
Flow diagram showing subject enrolment and exclusion.

The clinical US assessment of the IJVs and the invasive CVP measurement were completed in all 58 enrolled patients. The post-processing phase revealed a number of technical imperfections and/or subject related abnormalities (ECG trace or CVP images not recorded, n = 6; IJV-CSA edges not perfectly outlined, n = 4; CVP images not readable, n = 3; abnormal ECG, n = 5; abnormal IJV-CSA size, n = 6), which potentially could compromise model development. These traces were therefore deemed to be technically imperfect, and so the US video clips and CVP images from the 24 affected subjects were excluded from the post-processing phase in order to obtain a reliable dataset to analyse. In addition, in order to reduce the amount of data, only the right IJV US video clips were considered for this study. The right IJV was selected because this vessel is closer to the heart than the left IJV and thus is the dominant side in 80% of individuals. The clinical characteristics of the remaining 34 subjects are summarized in Table 1. Video clips and images from the remaining 34 subjects (age = 60 ± 12 years; 10 males/24 females) were finally processed and analysed (Fig 2). Sample results obtained during the post-processing phase are shown in S1 Table.

**Table 1:** Clinical characteristics of subjects.

Age (ys)	60±11
Male/female ratio	10/24
*Main diseases*, *n (%)*	
Neoplasia	34 (100%)
Coronary artery disease	3 (9%)
Thyroid diseases	4 (12%)
Hypertension	7 (21%)
Dyslipidaemia	3 (9%)
Diabetes	3 (9%)
*Main drugs*, *n (%)*	
Antihypertensives	7 (21%)
Statins	3 (9%)
Levothyroxine	4 (12%)
Corticosteroids	5 (15%)
Insulin	2 (6%)
Allopurinol	3 (9%)
Morphine/oxycodone	2 (6%)

Values are given as means ± SD for continuous variables and numbers for categorical variables.

**Fig 3:**
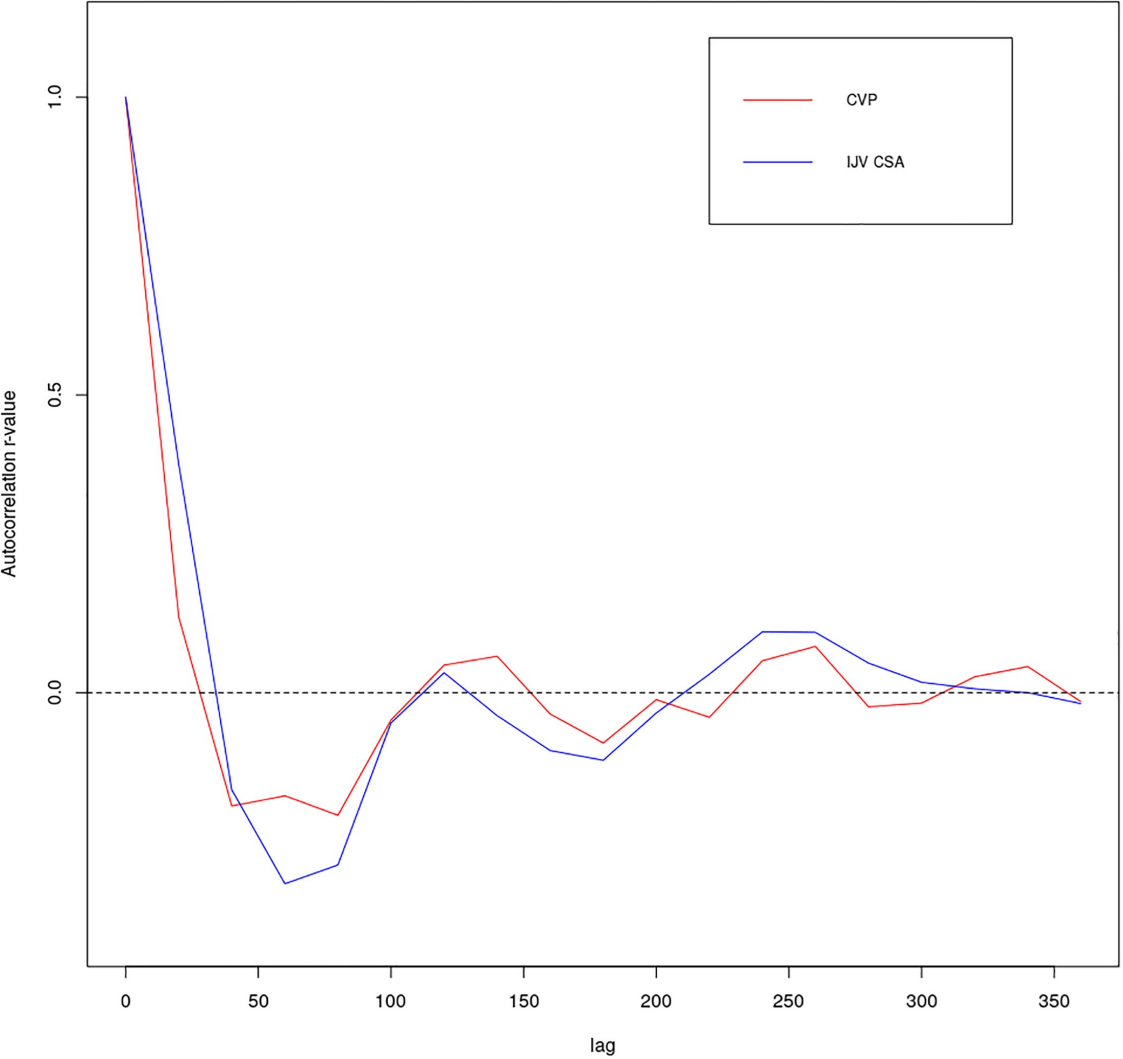
Mean ensemble autocorrelograms for the CVP and IJV CSA pulse signals (aggregated for all 34 subjects).

**Fig 4:**
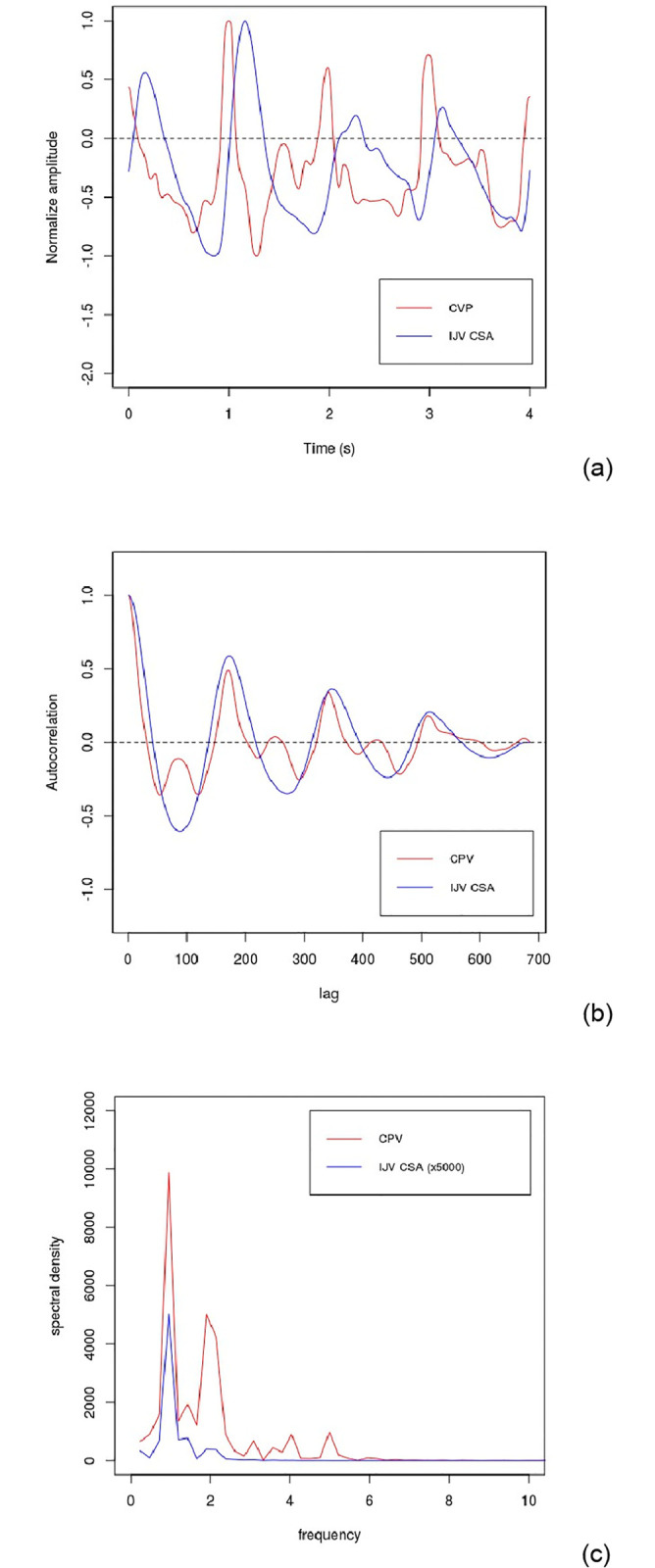
(a) Normalized time series signals; (b) autocorrelation signals; and (c) periodogram of the CVP and IJV CSA pulses for Subject 19.

The results of the autocorrelation analysis are presented in Table 2 and in Fig 3, while the spectral analysis results are presented in S2 and S3 Tables. Collectively, these reveal that while the CVP and IJV-CSA time series signals were poorly correlated (mean r = -0.018, SD = 0.357), their autocorrelation counterparts were highly positively correlated (mean r = 0.725, SD = 0.215) (Table 3). The poor correlation between the time series signals was in part due to the fact that the IJV-CSA signal lagged behind the CVP signal by on average 0.241 s (SD = 0.175 s) (Table 3), whereas in the autocorrelation signals were generally aligned because both pulses exhibited similar spectral characteristics (S2 and S3 Tables). This is well illustrated in Fig 4, which shows: (a) the normalized time series signals; (b) the autocorrelation signals; and (c) the combined periodogram, relating to Subject 19. From the periodogram in Fig 4(c) it can be seen that while both signals are dominated by a strong peak at 0.95 Hz, the CVP pulse exhibits a strong second peak at about 1.90–2.14 Hz, which is much weaker in the IJV-CSA pulse. This difference is reflected in the respective autocorrelation signals in Fig 4(b), which reveal that although both signals have the same general periodicity (indicating the dominance of a single frequency in both signals), the CVP autocorrelation signal is more complex, indicating that it is affected to a greater extent by other additional frequencies.

**Table 2:** Descriptive and aggregated autocorrelation results for both the central venous pressure (CVP) and internal jugular vein cross-sectional area (IJV-CSA) signals.

Parameter	CVP (n)	CVP (mean)	CVP (sd)	CVP (min)	CVP (max)	IJV-CSA (n)	IJV-CSA (mean)	IJV-CSA (sd)	IJV-CSA (min)	IJV-CSA (max)
Mean (cmH_2_O & cm^2^)	34	5.998	2.874	1.066	10.896	34	0.984	0.497	0.193	1.908
sd (cmH_2_O & cm^2^)	34	1.21	0.462	0.554	2.173	34	0.05	0.03	0.008	0.114
Median (cmH_2_O & cm^2^)	34	6.002	2.893	0.758	10.819	34	0.979	0.497	0.185	1.897
Min (cmH_2_O & cm^2^)	34	3.422	3.355	-2.271	9.664	34	0.879	0.466	0.125	1.813
Max (cmH_2_O & cm^2^)	34	8.667	2.660	3.34	12.739	34	1.090	0.525	0.287	2.040
lag0 (r-value)	34	1.000	0.000	1.000	1.000	34	1.000	0.000	1.000	1.000
lag20 (r-value)	34	0.127	0.339	-0.428	0.664	34	0.384	0.314	-0.429	0.841
lag40 (r-value)	34	-0.19	0.214	-0.549	0.264	34	-0.163	0.344	-0.830	0.542
lag60 (r-value)	34	-0.173	0.270	-0.636	0.587	34	-0.321	0.307	-0.784	0.602
lag80 (r-value)	34	-0.206	0.284	-0.64	0.731	34	-0.290	0.283	-0.656	0.642
lag100 (r-value)	34	-0.046	0.361	-0.572	0.727	34	-0.051	0.414	-0.643	0.682
lag120 (r-value)	34	0.046	0.354	-0.459	0.655	34	0.033	0.459	-0.723	0.767
lag140 (r-value)	34	0.061	0.263	-0.425	0.675	34	-0.039	0.397	-0.709	0.742
lag160 (r-value)	34	-0.036	0.304	-0.45	0.675	34	-0.097	0.332	-0.590	0.663
lag180 (r-value)	34	-0.085	0.250	-0.585	0.363	34	-0.114	0.314	-0.557	0.549
lag200 (r-value)	34	-0.012	0.265	-0.466	0.562	34	-0.034	0.316	-0.547	0.616
lag220 (r-value)	34	-0.041	0.254	-0.473	0.534	34	0.031	0.299	-0.583	0.632
lag240 (r-value)	34	0.054	0.250	-0.501	0.544	34	0.102	0.322	-0.459	0.674
lag260 (r-value)	34	0.078	0.279	-0.372	0.734	34	0.101	0.319	-0.370	0.835
lag280 (r-value)	34	-0.024	0.226	-0.349	0.552	34	0.050	0.287	-0.449	0.717
lag300 (r-value)	34	-0.018	0.197	-0.360	0.411	34	0.017	0.263	-0.425	0.574
lag320 (r-value)	34	0.027	0.237	-0.379	0.54	34	0.007	0.266	-0.420	0.566
lag340 (r-value)	34	0.044	0.193	-0.312	0.447	34	0.000	0.246	-0.387	0.597
lag360 (r-value)	34	-0.015	0.152	-0.429	0.304	34	-0.018	0.219	-0.494	0.483

CVP—central venous pressure; IJV-CSA—internal jugular vein cross-sectional area; n—number of observations; mean—mean value; sd—standard deviation; min—minimum value; max—maximum value.

**Table 3:** Correlations and lag times between the central venous pressure (CVP) and internal jugular vein cross-sectional area (IJV-CSA) signals for the respective subjects.

Subject ID	Correlation between the measure signals (r-value)	Correlation between the autocorrelation signals (r-value)	Incremental lag between the measure signals	Sampling interval (s)	Lag.time between the measure signals (s)
1	0.877	0.992	-4.000	0.009	-0.035
4	-0.286	0.956	-37.000	0.007	-0.261
5	0.501	0.947	-12.000	0.008	-0.094
8	-0.004	0.561	-55.000	0.009	-0.478
10	0.205	0.659	-13.000	0.010	-0.130
15	-0.003	0.528	-88.000	0.009	-0.759
18	-0.204	0.680	-34.000	0.008	-0.262
19	-0.013	0.821	-29.000	0.006	-0.170
20	-0.239	0.550	-53.000	0.009	-0.491
23	-0.388	0.509	-31.000	0.007	-0.220
24	-0.284	0.229	-69.000	0.010	-0.690
25	-0.196	0.789	-17.000	0.010	-0.165
28	0.682	0.929	-1.000	0.008	-0.008
30	0.062	0.224	-35.000	0.004	-0.134
31	-0.052	0.679	-41.000	0.004	-0.161
34	0.096	0.771	-17.000	0.007	-0.120
38	-0.592	0.826	-30.000	0.009	-0.278
39	-0.366	0.787	-37.000	0.012	-0.430
40	-0.075	0.732	-28.000	0.008	-0.219
42	-0.150	0.927	-44.000	0.004	-0.173
44	-0.408	0.855	-40.000	0.006	-0.245
46	0.018	0.339	-22.000	0.008	-0.171
47	-0.686	0.930	-32.000	0.013	-0.400
48	-0.102	0.969	-17.000	0.016	-0.270
49	-0.149	0.729	-45.000	0.010	-0.455
50	-0.354	0.870	-34.000	0.008	-0.283
51	0.677	0.926	-17.000	0.005	-0.077
52	0.115	0.916	-51.000	0.004	-0.179
53	-0.112	0.584	-52.000	0.003	-0.156
54	0.481	0.957	-18.000	0.006	-0.112
55	0.342	0.726	-7.000	0.003	-0.024
56	0.219	0.803	-31.000	0.005	-0.150
57	-0.059	0.583	-42.000	0.003	-0.119
58	-0.175	0.373	-64.000	0.004	-0.262
mean	-0.018	0.725	-33.735	0.007	-0.241
sd	0.357	0.215	19.181	0.003	0.175

mean—mean value; sd—standard deviation.

**Fig 5:**
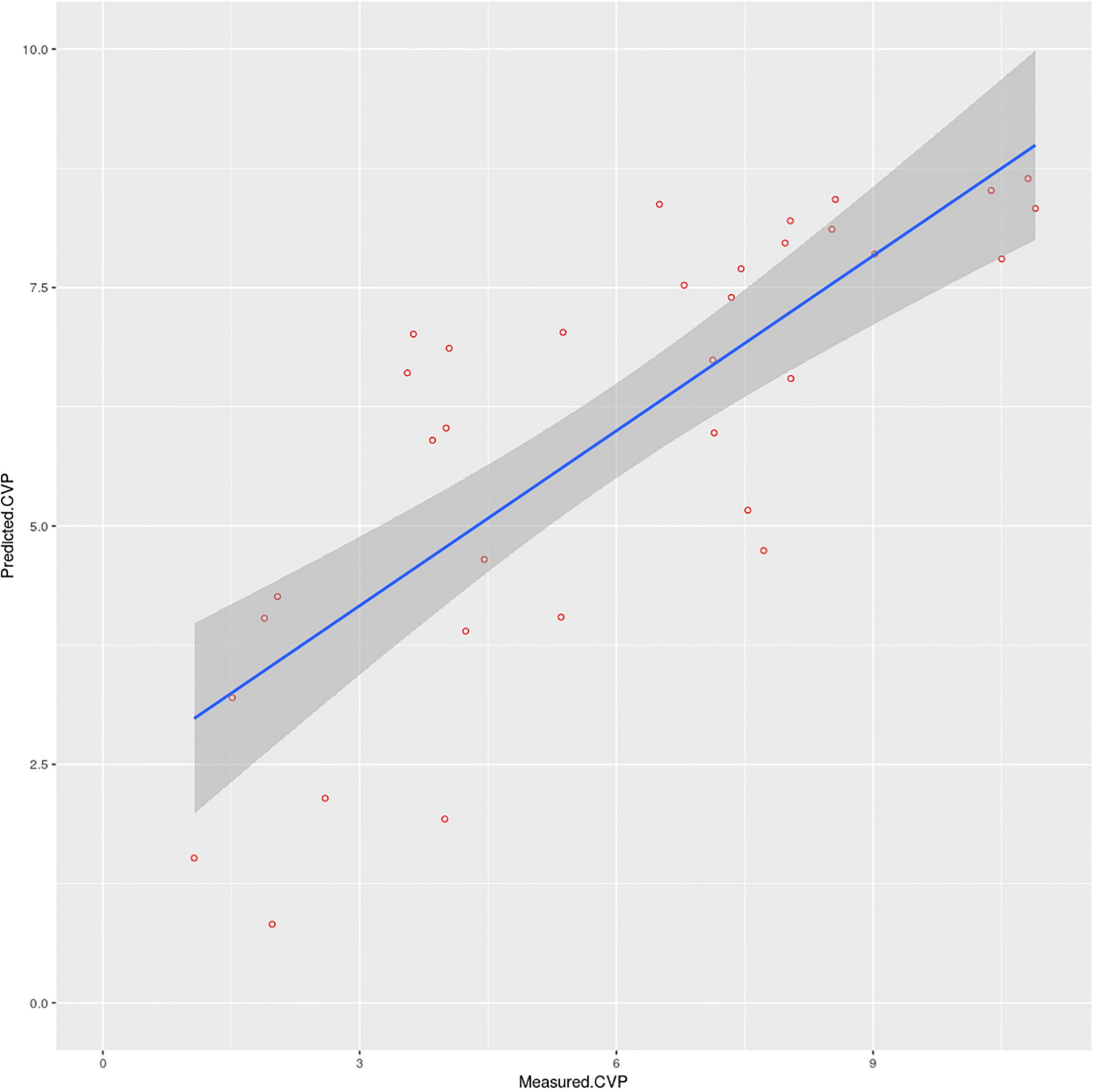
Regression scatter plot showing predicted and measured mean CVP values for the 34 subjects (units are in cmH_2_O).

The refined regression model results are presented in Table 4 and in Fig 5. This suggests that the simple algorithm shown in Eq 3 can be used to predict mean CVP with reasonable accuracy (r^2^ = 0.612) using just the mean IJV-CSA value and selected autocorrelation r-values as predictors. Post-hoc analysis revealed Cohen’s *f*^2^ to be 1.577 with a statistical power of 0.993.

$$\begin{array}{l} CVP_{pred}=(2.836\times CSA_{mean})-(32.469\times lag_{40})-(22.015\times lag_{80})-(7.590\times lag_{100})-\\ (18.417\times lag_{120})-(14.016\times lag_{140})-(23.054\times lag_{180})-(11.470\times lag_{220})-\\ (5.513\times lag_{240})-(8.478\times lag_{280})-(7.059\times lag_{320})-10.014 \end{array}$$ (3)

Where: *CVP*_*pred*_ is the predicted CVP; *CSA*_*mean*_ is the mean IJV-CSA; and lag 20–340 are the r-values for the specified lags extracted from the autocorrelation signal. This linear model predicted the mean CVP for the respective subjects (Fig 4) with a mean-absolute-error (mae) of 1.455 cmH_2_O, which rose to 2.436 cmH_2_O when 10-fold cross-validation was performed.

**Table 4:** Refined multiple linear regression model with mean central venous pressure (CVP) as the response variable.

Response Variable	Predictor Variables	Coefficient b (95% CI)	Significance p value	Model AIC (p value)	Model r^2^ (mae)	10-fold cross-validation r^2^ (mae)
CVP	Intercept	-10.014 (-19.73–-0.30)	0.044	161.1 (0.011)	0.612 (1.455)	0.498 (2.436)
	lag40	-32.469 (-53.43–-11.50)	0.004			
	Lag80	-22.015 (-38.27–-5.76)	0.010			
	Lag100	-7.590 (-14.66–-0.51)	0.037			
	Lag120	-18.417 (-28.41–-0.42)	0.001			
	Lag140	-14.016 (-25.85–-2.18)	0.022			
	Lag180	-23.054 (-36.28–-9.83)	0.002			
	Lag220	-11.470 (-19.95–-2.99)	0.010			
	Lag240	-5.513 (-12.11–1.09)	0.097			
	Lag280	-8.478 (-14.88–-2.87)	0.012			
	Lag320	-7.059 (-12.71–-1.41)	0.017			
	CSA mean	2.836 (0.93–4.74)	0.005			

With regards to clinical classification, the model correctly identified 26 out of 27 subjects (sensitivity = 96.3%) as having normal CVP; 0 out of 1 subject (sensitivity = 0.0%) as having a high CVP; and 3 out of 6 subjects (sensitivity = 50.0%) as having a low CVP. The overall classification accuracy of the model was 85.3% (Kappa = 0.472).

## Discussion

The jugular venous pulse is a physiological and pivotal parameter to describe cardio-circulatory function, as well as cardiac preload. In current clinical practice, it is a parameter that over time has been abandoned due to difficulty in creating reliable measurements when physically examining patients. The corresponding invasive ‘gold standard’, CVC, and the simultaneous diffusion of echocardiography, have in fact greatly reduced its use in clinical practice. However, CVC, being an invasive technique, has the great disadvantage that it is associated with an increased risk of clinical complications [8]. Therefore, the creation of a model that allows CVP to be detected quickly, reliably and non-invasively represents an important development, one that has relevance to several clinical applications, including the management of heart failure. In particular, it may be useful when monitoring drug therapy over time, something that cannot easily be done using echocardiography or an invasive manoeuvre. Therefore, having a rapid ultrasonographic test, which can also be performed by the general practitioner, could be decisive in the global management of heart failure, a disease that is thought to affect approximately 1–2% in the western world, with an incidence rate approaching 5–10 per 1000 persons per year [31], and which is more prevalent amongst the elderly and more common in women [32]. In addition, the non-invasive model could also be helpful in the evaluation of suspected hypovolemia, and cerebral venous return, or even in space medicine [21].

From the analysis presented above it can be seen that by utilizing the autocorrelation plot of the IJV-CSA pulse it is possible to predict mean CVP with reasonable accuracy (r^2^ >0.6) using a simple linear model. As such, we have been able to show that there is a clear relationship between the mean of the CVP pulse and the autocorrelation signal of the IJV pulse, as suggested by Eq 2. Having said this, we were somewhat surprised that the mean CVP could be estimated with such accuracy using just a simple linear model, given that the autocorrelation signal itself is non-linear. Indeed, it was only possible to use a linear model because the CSA autocorrelation signals were dominated by a cardiac frequency of just under 1 Hz, which meant that the CSA autocorrelation signals tended to exhibit a similar periodicity, as did many of the CVP autocorrelation signals (as exemplified in Fig 4(b)). Consequently, there was a broadly linear relationship between the extracted r-values for the respective lags and the CVP mean values for the study cohort, which we were able to exploit.

While Fourier analysis is frequently used to establish the spectral characteristics of blood vessels, in practice the frequency domain results produced can be difficult to interpret. By comparison, we found the autocorrelation signals much easier to interpret because they captured the ‘combined effect’ of all the frequencies acting on the respective vessels. Thus, we were able to quickly assess the similarities and dissimilarities between the respective signals using just their autocorrelations, something, which would be more difficult to achieve using either the time domain or frequency domain plots. For example, from Fig 4(b) it can be seen that although the CVP autocorrelation signal for Subject 19 is influenced by a strong secondary frequency of approximately 2 Hz (according to the periodogram in Fig 4(c)), this does not affect the overall periodicity of the CVP autocorrelation signal, which is the same as that of the CSA autocorrelation signal—something that is difficult to determine using either the respective time domain (Fig 4(a)) or frequency domain (Fig 4(c)) plots. As such, this suggests that autocorrelation plots have considerable potential when comparing venous haemodynamic signals.

Although in this initial proof-of-concept study we succeeded in producing an algorithm that can approximately predict mean CVP from the IJV-CSA pulse, we are aware that despite achieving an overall accuracy of 85%, the Kappa statistic was only 0.472, indicating just moderately good performance when clinically classifying patients. However, this was achieved using a simple linear model and a relatively small cohort, and it is anticipated that it should be possible in future studies to improve the predictive accuracy of the model by utilising a sophisticated machine learning strategy, such as a random forest [33]. Notwithstanding this, our results are promising when compare with those achieved using existing methodologies. For example, CVP has traditionally been assessed through visual inspection of changes in volume of the right IJV, when the upper body is inclined, using the sternal angle as the reference point. However, the IJV is often obscured by the overlying neck tissues making clear visual identification difficult, particularly for the less experienced examiner [34]. Consequently, the accuracy of the visual inspection technique has been shown to be little better than about 50–60% [35, 36]. It should also be noted that substantial variations can occur in observed CVP due to errors in transducer placement when taking invasive catheter measurements [37]. In the light of this, the methodology presented here appears to have merit and potentially could be extremely helpful particularly in emergency situations due to the speed with which vital information can be obtained non-invasively using a simple ultrasound investigation that can be easily performed at the patients’ bedside. Moreover, by utilising the model as a classification tool split into three categories (normal, high and low CVP), it is possible to facilitate its applicability to clinical practice.

One major limitation of out study is that the model was only tested on individuals with no signs or symptoms of: chronic or acute heart failure; arrhythmias; or relevant stenosis/insufficiency of the cardiac valves. Thus, we recommend that in future studies the applicability of the model to patients with concurrent heart diseases be studied as these might influence the behaviour of the algorithms used in the model. Also, because the model was produced using a relatively small study cohort, we recommend that further studies be undertaken using a larger cohort to confirm our findings and establish the sensitivity of the model. In addition, we are conscious that in this study we only used data from the right IJV and it may be that utilising both IJVs might produce superior results. However, none of our patients was affected by tricuspid regurgitation or pulmonary hypertension. It is therefore recommended that future work should explore both JVPs and their relationship with CVP in more challenging clinical conditions. Finally, future studies should evaluate the US-JVP methodology against alternative strategies such a NIRS.

In conclusion, we have been able to show that the autocorrelation signals of the respective CVP and IJV-CSA pulses exhibit marked similarities, and that the lagging r-values of the autocorrelation of the IJV-CSA pulse can be used to estimate mean CVP with reasonable accuracy. This novel approach appears to have considerable clinical potential, because it allows CVP to be measured non-invasively using ultrasonic assessment of the JVP. As such, the technique may have potential as a clinical monitoring and diagnostic tool.

## Supporting information

S1 Raw data (XLS)

S1 Table Mean and standard deviations of the internal jugular vein cross-sectional area (IJV-CSA) and central venous pressure (CVP) signals for each of the respective subjects. (DOC)

S2 Table Top five spectral frequencies, together with amplitudes, for the central venous pressure (CVP) signals from the respective subjects. (DOC)

S3 Table Top five spectral frequencies, together with amplitudes, for the internal jugular vein cross-sectional area (IJV-CSA) signals from the respective subjects. (DOC)

S1 Checklist TREND statement checklist. (PDF)

S1 Protocol (DOCX)

S2 Protocol (DOCX)
