# Supplementary material for: Central venous pressure estimation from ultrasound assessment of the jugular venous pulse
Source: PLoS One. 2020 Oct 28;15(10):e0240057. doi: 10.1371/journal.pone.0240057 (PMC7592775; doi:10.1371/journal.pone.0240057)
Supplement: S1 Table — (DOC) [file pone.0240057.s002.doc]

Table S1. Mean and standard deviations of the internal jugular vein cross-sectional area (IJV-CSA) and central venous pressure (CVP) signals for each of the respective subjects.

| Subject ID | IJV-CSA (mean)  (cm^2^) | IJV-CSA (st.dev)  (cm^2^) | CVP (mean)  (cmH_2_O) | CVP (st.dev)  (cmH_2_O) |
| --- | --- | --- | --- | --- |
| 1 | 1.73 | 0.06 | 4.0 | 0.6 |
| 4 | 0.19 | 0.04 | 4.0 | 2.2 |
| 5 | 1.62 | 0.10 | 10.9 | 0.7 |
| 8 | 0.27 | 0.01 | 2.0 | 1.4 |
| 10 | 1.50 | 0.10 | 10.4 | 0.7 |
| 15 | 0.36 | 0.01 | 1.1 | 1.5 |
| 18 | 1.45 | 0.05 | 8.0 | 0.9 |
| 19 | 0.48 | 0.05 | 1.5 | 2.1 |
| 20 | 1.50 | 0.01 | 5.4 | 0.8 |
| 23 | 1.91 | 0.06 | 3.6 | 1.7 |
| 24 | 1.35 | 0.07 | 1.9 | 0.8 |
| 25 | 0.61 | 0.04 | 6.8 | 1.3 |
| 28 | 1.40 | 0.07 | 7.7 | 1.5 |
| 30 | 0.43 | 0.08 | 8.1 | 1.2 |
| 31 | 1.14 | 0.04 | 8.6 | 1.9 |
| 34 | 0.42 | 0.06 | 7.3 | 1.0 |
| 38 | 0.97 | 0.04 | 4.2 | 1.1 |
| 39 | 0.52 | 0.02 | 10.8 | 1.1 |
| 40 | 1.68 | 0.11 | 7.5 | 1.1 |
| 42 | 0.55 | 0.06 | 5.4 | 2.0 |
| 44 | 0.45 | 0.02 | 2.6 | 1.0 |
| 46 | 1.26 | 0.05 | 8.0 | 1.2 |
| 47 | 0.96 | 0.01 | 6.5 | 1.4 |
| 48 | 0.27 | 0.03 | 2.0 | 0.7 |
| 49 | 1.22 | 0.02 | 4.0 | 1.6 |
| 50 | 1.29 | 0.08 | 9.0 | 1.7 |
| 51 | 0.92 | 0.03 | 10.5 | 0.7 |
| 52 | 0.62 | 0.10 | 3.4 | 1.6 |
| 53 | 0.89 | 0.02 | 8.5 | 1.3 |
| 54 | 1.27 | 0.10 | 7.1 | 0.7 |
| 55 | 1.37 | 0.09 | 7.1 | 1.0 |
| 56 | 0.45 | 0.05 | 3.9 | 1.1 |
| 57 | 1.37 | 0.06 | 4.4 | 1.4 |
| 58 | 1.06 | 0.02 | 7.5 | 0.6 |
