# Supplementary material for: Central venous pressure estimation from ultrasound assessment of the jugular venous pulse
Source: PLoS One. 2020 Oct 28;15(10):e0240057. doi: 10.1371/journal.pone.0240057 (PMC7592775; doi:10.1371/journal.pone.0240057)
Supplement: S2 Table — (DOC) [file pone.0240057.s003.doc]

Table S2 . Top five spectral frequencies, together with amplitudes, for the central venous pressure (CVP) signals from the respective subjects.

| ID | freq1  (Hz) | freq2  (Hz) | freq3  (Hz) | freq4  (Hz) | freq5  (Hz) | amp1 | amp2 | amp3 | amp4 | amp5 |
| --- | --- | --- | --- | --- | --- | --- | --- | --- | --- | --- |
| 1 | 0.958 | 1.118 | 2.076 | 1.278 | 1.917 | 130.725 | 19.381 | 13.087 | 9.425 | 5.919 |
| 4 | 0.986 | 2.958 | 0.657 | 1.150 | 1.479 | 2608.602 | 469.122 | 179.065 | 157.531 | 133.526 |
| 5 | 0.948 | 1.896 | 1.185 | 2.133 | 4.978 | 88.050 | 70.490 | 50.021 | 41.965 | 5.327 |
| 8 | 1.917 | 2.156 | 1.198 | 0.958 | 0.719 | 285.491 | 190.445 | 112.469 | 105.511 | 48.472 |
| 10 | 1.975 | 0.988 | 2.222 | 0.741 | 2.963 | 94.472 | 32.063 | 13.583 | 8.565 | 7.509 |
| 15 | 0.967 | 4.060 | 0.773 | 3.867 | 1.160 | 427.321 | 234.308 | 109.970 | 81.576 | 73.336 |
| 18 | 1.204 | 0.963 | 1.926 | 1.685 | 0.722 | 74.157 | 67.367 | 59.256 | 37.370 | 34.010 |
| 19 | 0.950 | 1.900 | 2.138 | 1.425 | 0.713 | 989.424 | 503.061 | 424.309 | 193.708 | 159.203 |
| 20 | 3.000 | 0.937 | 1.125 | 5.062 | 4.875 | 78.933 | 64.475 | 45.593 | 29.807 | 23.784 |
| 23 | 1.958 | 2.937 | 4.896 | 0.979 | 1.224 | 443.382 | 213.979 | 211.566 | 200.645 | 132.359 |
| 24 | 1.975 | 0.988 | 0.741 | 3.951 | 2.222 | 116.426 | 17.488 | 13.629 | 10.315 | 9.801 |
| 25 | 2.098 | 1.907 | 0.954 | 3.052 | 1.144 | 243.022 | 159.007 | 152.962 | 81.774 | 79.058 |
| 28 | 0.984 | 1.968 | 1.230 | 0.738 | 2.952 | 492.087 | 177.601 | 168.623 | 86.049 | 65.779 |
| 30 | 1.794 | 2.936 | 5.057 | 6.036 | 3.099 | 168.697 | 103.044 | 84.778 | 56.923 | 50.737 |
| 31 | 0.996 | 1.992 | 2.988 | 0.498 | 4.980 | 1716.193 | 1515.273 | 787.793 | 177.381 | 169.870 |
| 34 | 1.972 | 0.493 | 0.986 | 2.137 | 1.150 | 319.074 | 163.039 | 141.472 | 26.264 | 19.335 |
| 38 | 1.920 | 0.960 | 2.080 | 1.120 | 4.000 | 244.440 | 195.750 | 179.503 | 68.650 | 34.860 |
| 39 | 0.956 | 2.070 | 1.115 | 1.911 | 1.274 | 259.842 | 110.580 | 89.921 | 70.452 | 22.908 |
| 40 | 1.991 | 0.996 | 0.853 | 1.280 | 0.711 | 296.446 | 286.069 | 177.903 | 84.391 | 50.429 |
| 42 | 1.976 | 0.988 | 2.963 | 2.117 | 3.951 | 3486.978 | 1592.950 | 909.529 | 212.939 | 160.715 |
| 44 | 0.978 | 1.956 | 2.119 | 1.141 | 4.075 | 297.493 | 242.956 | 72.943 | 35.527 | 35.184 |
| 46 | 1.935 | 2.096 | 2.257 | 0.967 | 1.774 | 401.248 | 204.712 | 89.530 | 74.421 | 52.388 |
| 47 | 0.988 | 1.975 | 0.494 | 1.152 | 0.658 | 580.910 | 160.580 | 37.338 | 20.914 | 17.757 |
| 48 | 0.984 | 0.492 | 1.641 | 0.656 | 1.312 | 138.688 | 15.610 | 5.594 | 3.252 | 2.576 |
| 49 | 1.980 | 0.990 | 2.310 | 2.145 | 3.960 | 836.408 | 321.489 | 106.623 | 50.856 | 46.803 |
| 50 | 0.988 | 1.975 | 3.951 | 0.823 | 1.811 | 869.237 | 218.643 | 206.380 | 150.589 | 120.960 |
| 51 | 0.987 | 2.960 | 1.316 | 0.658 | 3.947 | 124.152 | 22.582 | 21.491 | 20.199 | 16.161 |
| 52 | 0.990 | 1.979 | 1.319 | 2.969 | 1.649 | 759.538 | 504.922 | 360.520 | 327.999 | 131.321 |
| 53 | 1.998 | 0.999 | 2.331 | 4.995 | 6.327 | 824.219 | 316.540 | 94.664 | 92.471 | 74.685 |
| 54 | 0.994 | 1.988 | 2.981 | 0.745 | 0.497 | 182.178 | 24.561 | 18.237 | 13.210 | 8.839 |
| 55 | 0.653 | 0.980 | 1.960 | 1.633 | 1.307 | 289.366 | 137.075 | 80.719 | 69.909 | 53.546 |
| 56 | 0.994 | 1.987 | 4.968 | 2.981 | 0.331 | 286.873 | 257.847 | 36.859 | 34.223 | 32.540 |
| 57 | 1.956 | 2.281 | 2.607 | 1.630 | 1.304 | 844.797 | 519.071 | 258.122 | 232.484 | 205.475 |
| 58 | 1.952 | 0.976 | 2.603 | 0.325 | 0.651 | 142.200 | 32.155 | 16.893 | 9.521 | 8.156 |

Legend: amp1, ... amp5 – amplitudes of the top five spectral ‘frequencies’ in desending order of magnitude; freq1, ... freq5 – frequencies of the top five ‘frequencies’.
