# Supplementary material for: Central venous pressure estimation from ultrasound assessment of the jugular venous pulse
Source: PLoS One. 2020 Oct 28;15(10):e0240057. doi: 10.1371/journal.pone.0240057 (PMC7592775; doi:10.1371/journal.pone.0240057)
Supplement: S3 Table — (DOC) [file pone.0240057.s004.doc]

Table S3. Top five spectral frequencies, together with amplitudes, for the internal jugular vein cross-sectional area (IJV-CSA) signals from the respective subjects.

| ID | freq1 | freq2 | freq3 | freq4 | freq5 | amp1 | amp2 | amp3 | amp4 | amp5 |
| --- | --- | --- | --- | --- | --- | --- | --- | --- | --- | --- |
| 1 | 0.958 | 1.118 | 1.917 | 1.278 | 2.076 | 2.052 | 0.471 | 0.126 | 0.104 | 0.086 |
| 4 | 0.986 | 0.329 | 1.150 | 0.822 | 1.972 | 0.970 | 0.122 | 0.086 | 0.062 | 0.053 |
| 5 | 0.948 | 1.896 | 2.133 | 0.237 | 1.185 | 2.107 | 0.769 | 0.585 | 0.469 | 0.346 |
| 8 | 0.958 | 2.156 | 1.917 | 1.198 | 2.635 | 0.034 | 0.005 | 0.004 | 0.003 | 0.002 |
| 10 | 0.988 | 1.975 | 0.494 | 1.235 | 1.481 | 1.880 | 0.746 | 0.269 | 0.181 | 0.128 |
| 15 | 0.967 | 1.933 | 2.127 | 0.773 | 0.580 | 0.023 | 0.014 | 0.011 | 0.006 | 0.004 |
| 18 | 0.963 | 1.204 | 0.722 | 1.444 | 2.167 | 0.724 | 0.187 | 0.051 | 0.047 | 0.023 |
| 19 | 0.950 | 1.425 | 1.188 | 0.713 | 1.900 | 1.008 | 0.156 | 0.145 | 0.139 | 0.080 |
| 20 | 0.750 | 0.937 | 1.125 | 1.312 | 2.062 | 0.033 | 0.022 | 0.016 | 0.005 | 0.004 |
| 23 | 0.979 | 1.224 | 0.734 | 1.958 | 2.203 | 1.235 | 0.321 | 0.135 | 0.077 | 0.065 |
| 24 | 0.988 | 0.494 | 1.235 | 2.222 | 0.247 | 1.385 | 0.364 | 0.093 | 0.057 | 0.043 |
| 25 | 0.954 | 2.098 | 1.144 | 1.907 | 1.717 | 0.299 | 0.131 | 0.098 | 0.080 | 0.035 |
| 28 | 0.984 | 1.230 | 2.952 | 1.722 | 0.246 | 1.828 | 0.163 | 0.112 | 0.066 | 0.055 |
| 30 | 0.979 | 1.958 | 1.142 | 2.936 | 0.653 | 8.294 | 0.876 | 0.497 | 0.172 | 0.165 |
| 31 | 0.996 | 2.988 | 1.992 | 1.162 | 1.328 | 1.718 | 0.118 | 0.111 | 0.100 | 0.084 |
| 34 | 0.986 | 1.972 | 2.137 | 1.315 | 2.301 | 1.053 | 0.752 | 0.130 | 0.112 | 0.082 |
| 38 | 0.960 | 1.920 | 2.080 | 1.120 | 3.040 | 0.403 | 0.143 | 0.117 | 0.108 | 0.021 |
| 39 | 0.956 | 1.115 | 0.796 | 1.911 | 0.637 | 0.082 | 0.046 | 0.018 | 0.005 | 0.003 |
| 40 | 0.996 | 0.853 | 0.711 | 1.280 | 1.564 | 4.367 | 1.473 | 0.534 | 0.518 | 0.350 |
| 42 | 1.976 | 0.988 | 1.411 | 1.270 | 2.117 | 2.524 | 2.113 | 0.139 | 0.138 | 0.106 |
| 44 | 0.978 | 1.956 | 1.630 | 1.141 | 1.304 | 0.160 | 0.031 | 0.021 | 0.018 | 0.016 |
| 46 | 0.967 | 0.806 | 1.129 | 1.290 | 2.096 | 0.882 | 0.177 | 0.159 | 0.131 | 0.121 |
| 47 | 0.988 | 0.658 | 1.975 | 3.128 | 1.646 | 0.017 | 0.005 | 0.002 | 0.001 | 0.001 |
| 48 | 0.984 | 1.148 | 0.656 | 0.820 | 1.805 | 0.231 | 0.039 | 0.021 | 0.012 | 0.011 |
| 49 | 0.990 | 1.980 | 1.155 | 1.485 | 2.145 | 0.081 | 0.036 | 0.010 | 0.007 | 0.006 |
| 50 | 0.988 | 0.494 | 1.317 | 0.329 | 1.975 | 2.875 | 0.211 | 0.152 | 0.123 | 0.118 |
| 51 | 0.987 | 1.644 | 0.658 | 0.329 | 2.960 | 0.451 | 0.056 | 0.050 | 0.036 | 0.031 |
| 52 | 0.990 | 1.979 | 1.319 | 0.660 | 2.969 | 5.640 | 1.461 | 0.942 | 0.525 | 0.443 |
| 53 | 0.999 | 0.666 | 1.998 | 1.332 | 1.665 | 0.321 | 0.084 | 0.067 | 0.030 | 0.013 |
| 54 | 0.994 | 1.988 | 1.491 | 0.497 | 2.981 | 3.398 | 0.456 | 0.203 | 0.166 | 0.156 |
| 55 | 0.980 | 0.327 | 1.307 | 1.633 | 0.653 | 3.959 | 0.823 | 0.595 | 0.514 | 0.390 |
| 56 | 0.994 | 1.987 | 0.331 | 1.325 | 0.662 | 1.084 | 0.179 | 0.060 | 0.044 | 0.039 |
| 57 | 1.630 | 0.978 | 2.281 | 1.956 | 2.933 | 0.763 | 0.599 | 0.418 | 0.328 | 0.177 |
| 58 | 1.301 | 0.976 | 2.928 | 2.603 | 1.952 | 0.075 | 0.060 | 0.037 | 0.032 | 0.027 |

Legend: amp1, ... amp5 – amplitudes of the top five spectral ‘frequencies’ in desending order of magnitude; freq1, ... freq5 – frequencies of the top five ‘frequencies’.
