# Supplementary material for: Central venous pressure estimation from ultrasound assessment of the jugular venous pulse
Source: PLoS One. 2020 Oct 28;15(10):e0240057. doi: 10.1371/journal.pone.0240057 (PMC7592775; doi:10.1371/journal.pone.0240057)
Supplement: S1 Protocol — (DOCX) [file pone.0240057.s006.docx]

| 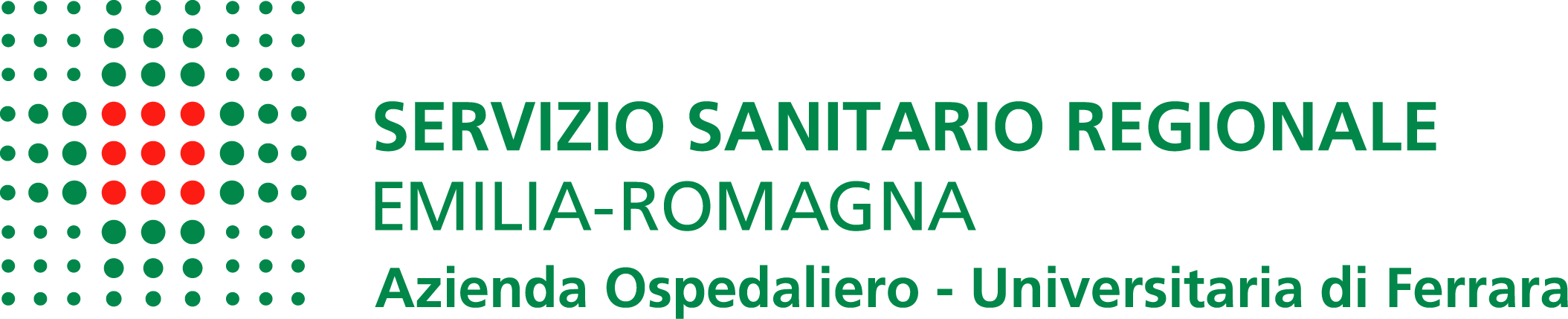 | 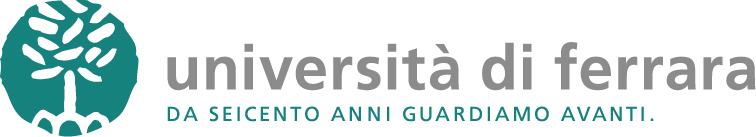 |
| --- | --- |

**OPERATIVE UNIT OF TRANSLATIONAL SURGERY**

*Director: Prof Paolo Zamboni*

**Study protocol (version 1)**

**Validation of a novel non-invasive technique based on real time ultrasound B-mode to assess the jugular venous pulse**

**OVERALL SUMMARY**

The jugular venous pulse (JVP) reflects changes in the right atrial pressure and its evaluation can be useful in managing many emergency conditions as well as in the diagnosis and/or prognosis of many heart and lung diseases. It is traditionally evaluated by a physical examination that visually analyzes the change in volume of the internal jugular vein (IJV) whilst also providing an estimate of the central venous pressure (CVP), though with a low accuracy. Furthermore, the real CVP is measured invasively through the cannulation of the venous system, thus not feasible as a routine approach. We hypothesized that it is possible to derive the JVP waveform through the ultrasound B-mode sequential assessment of the cross-sectional area of the IJV along the cardiac cycle. A preliminary pre-clinic study was performed to develop a proper method and to assess its accuracy and internal validity. The present project aims to fully implement and validate this new method and to test its transferability into a clinical setting.

**BACKGROUND AND STATE OF THE ART**

The evaluation of the jugular venous pulse (JVP), defined as the movement of expansion of the jugular veins due to changes in pressure in the right atrium, provides valuable information about cardiac hemodynamics and filling pressures (1), characteristic wave patterns pathognomic of cardiac diseases (2), and an indirect estimate of the central venous pressure (CVP). The JVP evaluation can be useful in managing many emergency conditions for guiding the fluid administration as well as in the diagnosis and/or prognosis of many heart (3) and lung diseases, notwithstanding it is often neglected by clinicians (2). JVP is traditionally evaluated by a physical examination that visually analyzes the change in volume of the internal jugular vein, with variable inclination of the upper body and the sternal angle as the reference point (4). However, it is considered a misunderstood and difficult physical technique (5) and the accuracy of the estimated CVP is no better than 50–60% (6). Besides, the real CVP is measured invasively through the cannulation of the venous system, thus not feasible as a routine approach. Several methods have been reported aiming to measure JVP and CVP either non-invasively or in a minimally invasive approach (6-9) but none showed enough accuracy and precision (6), neither completeness of information provided for clinicians, nor ease of use to enter into the routine clinical practice.

We therefore hypothesized that it is possible to derive the JVP waveform through the ultrasound B-mode sequential assessment of the cross-sectional area of the IJV along the cardiac cycle. A preliminary pre-clinic study had already been performed in collaboration with the Department of Physics of the University of Ferrara to develop the technical feasibility of this evaluation and to assess its accuracy and internal validity (10). Such methodology would also allow, through an algorithm and software, the ability to derive and analyze the waveform in post-processing, whilst already at the bedside, minimizing the evaluation time required and reducing the possible technical difficulties for the operator. The present project aims to fully implement and validate this new method and to test its transferability into a clinical setting.

**AIM OF THE STUDY**

**AIM 1**: To validate in a clinical setting a novel non-invasive technique based on real time ultrasound B-mode to assess the JVP (bed-side post processing analysis).

**AIM 2**: To verify if the proposed method can be transferred from our hemodynamics and fluid dynamics lab to the clinical setting, by using any kind of US equipment widely available in any hospital in our Country, and implemented by operators without a specific qualification in US technique, but after appropriate training.

**EXPERIMENTAL DESIGN AIM 1**

Aim 1 will be divided into 2 phases.

Phase 1: improvement and refinement of US-JVP software usability.

In a preliminary experiment (10) the variation of the cross-sectional area of the IJV during the cardiac cycle by means of real time B-mode images sequence was studied, and a resultant waveform trough time was outlined. It was also verified if an automatic procedure could reliably substitute a time inefficient operator tracing. Thanks to an algorithm and the development of a related original software, it was tested that the assessment of this waveform can be performed in post-processing analysis. This first phase of the project will be devoted:

a) To improve the usability of the algorithm and related software.

b) To study the relationship between variations of IJV cross sectional area and variations of IJV inside pressure in order to develop a physical model providing an estimate of the CVP.

c) To study the different waveforms derived from the US-JVP outlining a pool of normal or potential diagnostic abnormal patterns.

This will lead to further refinement of the algorithm and the related software in view of the Phase 2 of the project and the final validation of the US-JVP technique. This first phase will be carried out by close collaboration among physicists and clinicians.

Setting

OU of Translational Surgery and OU of University Anesthesiology and Intensive care, University Hospital of Ferrara and Eco-Fluid dynamics lab of the Department of Physics, University of Ferrara.

Subjects

At least n=80 consecutive healthy adult subjects of different sex and age will be studied by means of US-JVP technique. This number of subjects will likely allow us to detect and to evaluate the different morphological subtypes of IJVs.

Inclusion criteria: adult subjects (> 18 years).

Exclusion criteria: current acute disease, chronic cardiac or lung diseases, neurodegenerative diseases, pregnancy.

At least n=50 consecutive spontaneously breathing hospitalized patients requiring a scheduled, or with deferred emergency, measurement of CVP by direct cannulation, will be also studied at bedside by means of US-JVP. In particular, a portion of this sample (n=25) will include patients affected by various cardiac pathologies (e.g. congestive heart failure, atrial septal defect, tricuspid regurgitation, atrial fibrillation, tricuspid stenosis, AV blocks, constrictive pericarditis) in order to outline different US-JVP waveforms patterns with a potential diagnostic power.

Inclusion criteria: hospitalized adult > 18 years in spontaneous breathing requiring a direct measurement of the CVP, scheduled or in a deferred emergency. Part of this sample will include patients affected by cardiac diseases (e.g. congestive heart failure, atrial septal defect, tricuspid regurgitation, atrial fibrillation, tricuspid stenosis, AV blocks, constrictive pericarditis)

Exclusion criteria: no clinical exclusion criteria. Pregnancy.

The sample size has been determined considering the potential recruitable healthy subjects and patients referred to the intensive care unit, the workload actually sustainable by the investigators and the need for the scientific aim of the research (e.g. n=50 measurements will allow obtaining a JVP waveform derived from about 6000 tracing by hand in order to refine the algorithm that is the basis of the US-JVP software automatic procedure).

Methods

US-JVP acquisition imaging: all subjects, in supine position with the neck latch on the longitudinal axis and tilted 45° backward, will undergo US scanning (B-mode images sequence) of the right and left IJVs using a probe linear array 7.5-11 MHz. After the correct image acquisition, for each neck side a clip of 30” will be recorded. US assessments will be performed by a skilled operator blinded to clinical conditions of the hospitalized patients and to subsequent measures of invasive CVP.

Estimated total time for US assessment is 5’. Immediately afterwards, in hospitalized patients, CVP assessment by direct cannulation of the venous system will be performed by competent staff operators according to the standard.

Phase 2: Full validation process of the improved US-JVP measurements

The validation processes will include assessment of the diagnostic power of the US-JVP estimated CVP values and US-JVP waveforms, including evaluation of sensibility, specificity, predictive positive and negative values, accuracy and precision, compared to the gold standard of direct invasive measurement of CVP.

Setting

OU of Translational Surgery and OU of University Anesthesiology and Intensive care, University Hospital of Ferrara and Eco-Fluid dynamics lab of the Department of Physics, University of Ferrara.

Subjects

n= 150 consecutive hospitalized spontaneously breathing patients requiring a scheduled, or with deferred emergency, measurement of CVP by direct cannulation.

Inclusion criteria: hospitalized adult > 18 years in spontaneous breathing requiring a direct measurement of the CVP, scheduled or in a deferred emergency.

Exclusion criteria: no clinical exclusion criteria. Pregnancy,

Methods

All patients will undergo a US scanning of the neck at bedside as described above, performed independently and sequentially by two skilled operators to verify the inter-observer reliability. To verify the test-retest reliability, operators will repeat the procedure twice and two 30”clips will be recorded. Operators will be blinded to the respective evaluations, to the clinical condition of the patient and to the subsequent measures of invasive CVP. Immediately afterwards CVP assessment by direct cannulation of the venous system will be performed by competent staff operators according to the standard.

For all the phases of the study, the measurement of CVP will not be carried out for experimental purposes but will be part of the scheduled plan for each subject during the hospitalization. Only the numerical data obtained will be considered for the purpose of this study. Therefore, no variation is expected with respect to the normal diagnostic-therapeutic pathway already defined for each study subject.

**EXPERIMENTAL DESIGN AIM 2**

Aim 2 will be developed in 2 phases.

The transferability of the new technique will be tested in an emergency clinical setting and in a specialized one. Four clinicians without specific expertise in the use of ultrasound technique will be involved in this project. They will be asked to use the new US-JVP technique, before (Phase 3) and after specific training (Phase 4).

Setting

OU of Translational Surgery, OU Emergency Service and OU Neurosurgery University Hospital of Ferrara.

Subjects: n = 40 consecutive patients requiring an evaluation of the JVP /CVP in scheduled or referred emergency.

Inclusion criteria: patients requiring an evaluation of the JVP/CVP in deferred emergency.

Exclusion criteria: no clinical exclusion criteria. Pregnancy,

Methods

This second part of the study will involve n=4 clinicians belonging to emergency and specialized operative units, without a specific skill in US evaluations. Clinicians will receive a basic explanation about the US-JVP technique and its modality of imaging acquisition.

Phase 3: Each clinician will evaluate n=10 subjects using US-JVP technique (independently, in different times and each one in their own context). Measurements recorded (twice for each neck side for assessing repeatability and reproducibility) will be compared to those ones immediately collected afterwards by a skilled operator.

Phase 4: The whole experiment will be repeated by the same clinicians, after a specific detailed training on US-JVP technique, to evaluate any difference in accuracy and precision with respect to phase 3.

The ultrasound evaluation will be additional to the normal diagnostic-therapeutic pathway defined for the patient who will therefore not undergo any substantial variation.

**STATISTICAL ANALYSIS**

Aim1-Phase 1. Descriptive data will be expressed as the mean ± standard deviation or median and range according to the nature of the variables.

Aim1-Phase 2. The new US-JVP method will be compared to the gold standard of invasive CVP measurement in patients from an intensive care unit, with operators blinded to the patient’s condition and values of the direct measure of CVP. The number of patients required to estimate sensitivity and specificity, in a target population with a prevalence of 50%, assuming a clinically acceptable width of the 95% confidence interval of 10%, and a sensitivity and a specificity at least both of sizes 0.80, is 123. In order to correct for missing values, we will collect 20% patients more for a total sample size of 150 (11). Correlation between the two methods (US-CVP) will be analyzed using Pearson’s coefficient. The Bland-Altman method will be used to analyze the agreement between the measurements. The validation processes will assess the diagnostic power of the US-JVP evaluating sensitivity, specificity, predictive positive and negative values, accuracy and precision. Receiver operating characteristic (ROC) curves will be used to assess the sensitivity and specificity corresponding to different cutoffs for the new US-JVP method. Inter observers and test-retest reliability will be also calculated.

Aim 2 – Phase 3. The sample size needed to assess reliability between operators is based on Walter et al. (12). Assuming a minimally acceptable level of reliability of 0.60, given an alternative hypothesis of a level of reliability of 0.80, an alpha level of 0.05, a power of 0.80, a total of 40 patients are necessary to be evaluated independently by each clinician. Intraclass correlation coefficient (ICC) will be determined.

Aim 2 – Phase 4. Intraclass correlation coefficient (ICC) will be determined after the training. The Bland-Altman method will be also used to analyze the agreement between clinicians.

The statistical approach and analysis will be followed and carried out by statisticians of the Department of Public Health, Experimental and Forensic Medicine, Unit of Biostatistics and Clinical Epidemiology, University of Pavia

**RISK ANALYSIS, POSSIBLE PROBLEMS AND SOLUTIONS**

The main risk related to Aim1 is the failure of the statistical validation process of the new method compared to gold standard. For this reason, it has been decided to proceed to an *ad interim* analysis (at month 12). If data is not satisfactory, physicists will revise the engineering of the software and/or the precision of the physical model before continuing the experiment.

**SIGNIFICANCE AND INNOVATION**

It is known that the assessment of the JVP for its diagnostic and prognostic power is desirable, despite the fact that it is currently neglected (1-9). The main reason is the uncommon skill required for JVP clinical assessment (4,5). Alternatively, a non-invasive, rapid and at low cost instrumental technique able to comprehensively assess the JVP and CVP at bedside does not exist so far. Completely noninvasive methods have not been reported to have the accuracy and precision necessary for routine use and interchangeability with traditional invasively measured CVP (6). In addition, the technique here proposed, differently from other US methods previously developed to assess the JVP (6-9), might not only be able to provide an estimate value of CVP, but also to outline characteristic wave patterns pathognomic of cardiac diseases. This technique might be useful in a variety of multidisciplinary emergency and elective clinical situations.

**DESCRIPTION OF THE COMPLEMENTARITY AND SYNERGY RESEARCH TEAM**

The project includes a multidisciplinary team of clinicians, technicians, physicists and statisticians with a strong background in the specific topics covered, as well as a history of previous collaborations. Phase 1 will involve investigators expert in hemodynamics and US of the vascular system and physicists to develop a new non-invasive technique to assess the JVP.

Vascular medical expertise and applied physic skill are needed to fully implement the algorithm and the software supporting the new method. The invasive CVP assessments will be performed in an intensive care unit under the supervision of an accountable investigator. Aim 2, testing the transferability of the method, will be carried out in synergy among clinicians of emergency and specialized units and the experts in hemodynamics and US. Skilled US technicians will attend all the experimental phases for the correct images acquisition, as well the statisticians who will be responsible for a load-bearing part of the project.

**REFERENCES**

1. Naveen G et al. Jugular Venous Pulse : An Appraisal. JIACM, 2000;1(3):260-9.

2. Chua Chiaco JM et al. The jugular venous pressure revisited. Cleve Clin J Med 2013;80(10):638-44.

3. Drazner MH et al. Prognostic importance of elevated jugular venous pressure and a third heart sound in patients with heart failure. N Engl J Med. 2001;345(8):574-81.

4. Mackenzie J. The study of the pulse, arterial, venous and hepatic, and of the movements of the heart. Edinburgh: Young J. Pentland 1902.

5. Applefeld MM. The Jugular Venous Pressure and Pulse Contour. In Clinical Methods: The History, Physical, and Laboratory Examinations. 3rd edition. Boston: Butterworths; 1990.

6. Ward KR et al. A new noninvasive method to determine central venous pressure. Resuscitation. 2006;70(2):238-46.

7. Keller AS et al. Diagnostic accuracy of a simple ultrasound measurement to estimate central venous pressure in spontaneously breathing, critically ill patients. J Hosp Med 2009;4(6):350-5.

8. Deol GR et al. Ultrasound accurately reflects the jugular venous examination but underestimates central venous pressure. Chest 2011;139(1):95-100.

9. Lipton B. Estimation of central venous pressure by ultrasound of the internal jugular vein. Am J Emerg Med 2000;18(4):432-4.

10. Sisini F et al. A ultrasonographic technique to assess the jugular pulse, 2014 (submitted for peer review).

11. Buderer NMF. Statistical methodology: I. incorporating the prevalence of disease in the sample size calculation for sensitivity and specificity. Acad Emerg Med 1996; 3:895-900.

12. Walter SD, Eliasziw M, Donner A. Sample size and optimal designs for reliability studies Statistics in Medicine 1998, 17, 101-110.

13.

**Timeline/ Milestones**

-0-6 months period: Definitive implementation of the US-JVP technique (Aim 1-Phase1).

-6-18 months period: Full validation of the US-JVP technique (Aim 1-Phase 2).

-18-21 months period: Data analysis Aim 1.

-21-33 months period: Evaluation of the transferability of the US-JVP technique in a multidisciplinary clinical setting (Aim 2-Phase 3 and Phase 4).

-33-36 months period: Data analysis Aim 2 and dissemination of the overall scientific results.

**Milestones 18 month**

After a first phase (0-6 months) devoted to the full implementation of the US-JVP, in particular for the refinement of the software allowing the automatic evaluation of the JVP and of the physical model for the estimate of CVP, at 18 months we expect the conclusion of the experimental part of Aim 1, namely the validation of the new technique in a clinical setting.

**Milestones 36 month**

In the 18-21 months period the data analysis related to Aim 1 will be performed.

In the 18-33 months period we will carry on with Aim 2 of the project, specifically the verification of the operative transferability of the new method in clinical/emergency settings. The last step period (33-36 months) will be devoted to the final data analysis and dissemination of the overall scientific results.

**EQUIPMENT AND RESOURCES AVAILABLE**

Measurements of US-JVP will be performed by operators specifically trained in PhD programs devoted to the detection of non-conventional parameters of the brain circulation assessed by innovative US techniques. A portable Echo-Color Doppler instrument is available in the O.U. of the PI, while another analogue instrument will be acquired in leasing for the segment of the project at bedside in the other O.Us. Invasive measurements of the CVP will be performed as part of the usual clinical practice by skilled operators under the supervision of an accountable senior investigator. The Eco-Fluid dynamics lab of the Department of Physics-University of Ferrara (which is involved in the project as a scientific supporting part) is equipped with innovative technologies purposely created to study the blood flow in physiological and pathological conditions (e.g. hydraulic circuit that simulates the blood circulation in the neck) staffed by a team of researchers skilled in the analysis of the response of Echo-Doppler flow. The statistical analysis will be performed by the Department of Public Health, Experimental and Forensic Medicine, Unit of Biostatistics and Clinical Epidemiology, University of Pavia. In addition, all the investigators involved in the project have got a consolidated history of previous collaboration.

**TRANSLATIONAL RELEVANCE AND IMPACT FOR THE NATIONAL HEALTH SYSTEM**

In case of positive results, the new US-JVP technique could be proposed in clinical practice for rapid, non-invasive, and cost-effective evaluation of JVP and CVP by means of equipment widely available in the Hospitals of our Country, and by multidisciplinary operators appropriately trained. Cardiovascular US-JVP assessment could be useful in a variety of multidisciplinary emergency and elective clinical situations.
**ETHICAL ASPECTS**

To each patient, the investigator will describe the study in detail and read the information sheet allowing him to ask questions whenever a part is difficult to understand. Subsequently, he will be provided with the Consent Form which he is required to read before signing. Patient data will be processed in full compliance with the privacy protection law. They will then be recorded, processed and archived in order to guarantee their security and confidentiality and when included in publications and / or presentations at scientific conferences they will be subjected to statistical processing and then transformed into anonymous data.
