# Supplementary material for: Central venous pressure estimation from ultrasound assessment of the jugular venous pulse
Source: PLoS One. 2020 Oct 28;15(10):e0240057. doi: 10.1371/journal.pone.0240057 (PMC7592775; doi:10.1371/journal.pone.0240057)
Supplement: S2 Protocol — (DOCX) [file pone.0240057.s007.docx]

| 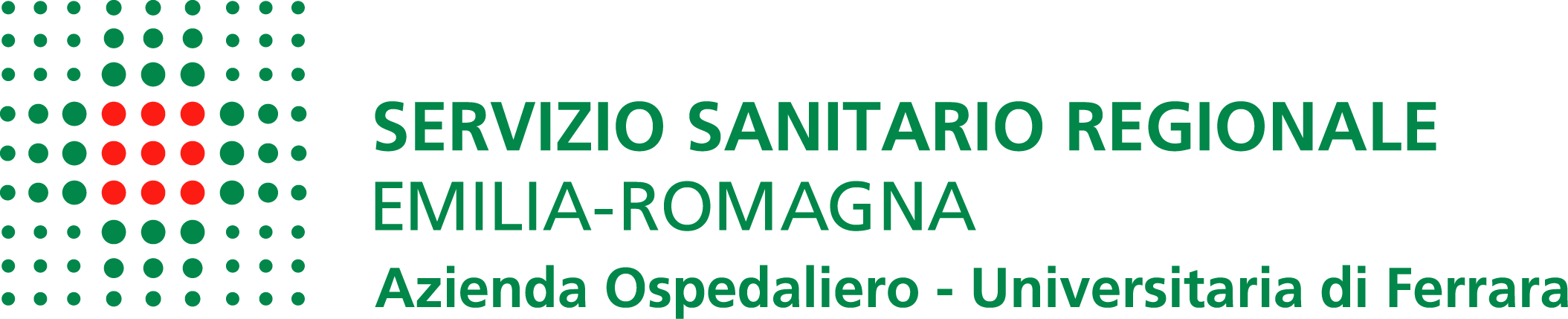 | 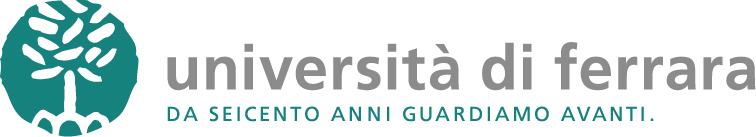 |
| --- | --- |

**UNITA’ OPERATIVA DI CHIRURGIA TRASLAZIONALE**

*Direttore: Prof Paolo Zamboni*

**PROTOCOLLO DELLO STUDIO (versione 1)**

**Validazione di una nuova metodica non invasiva basata sull’ultrasonografia B-mode in tempo reale per la valutazione del polso venoso giugulare**

**SOMMARIO**

Il polso venoso giugulare (PVG) riflette le variazioni pressorie dell’atrio destro e la sua valutazione risulta utile in condizioni di emergenza/urgenza così come nella diagnosi e/o prognosi di molti stati patologici cardiaci e polmonari. Il PVG è tradizionalmente valutato clinicamente visualizzando le variazioni di volume della vena giugulare interna. L’ispezione delle vene del collo fornisce anche una stima, sebbene poco accurata, della pressione venosa centrale (PVC). La misura precisa e diretta della PVC può avvenire solo in maniera invasiva attraverso cateterismo del sistema venoso, approccio pertanto specialistico e poco idoneo alla pratica clinica di routine. Si è ipotizzato di poter derivare il PVG mediante la tecnica ultrasonografica B-mode rilevando immagini sequenziali dell’area di sezione della vena giugulare interna nel corso di un ciclo cardiaco. E’ già stato condotto uno studio pre-clinico preliminare per mettere a punto una metodica di rilevazione appropriata e valutarne accuratezza e validità interna. Il presente progetto ha lo scopo di completare lo sviluppo e la validazione di questa nuova metodica e di sperimentarne la trasferibilità in un contesto clinico.

**BACKGROUND E STATO DELL’ARTE**

La valutazione del PVG, definito come il movimento di espansione delle vene giugulari dovute alle variazioni pressorie nell’atrio destro, è in grado di dare informazioni sull’emodinamica cardiaca e le pressioni di riempimento (1), definire le forme d’onda patognomoniche di patologia cardiaca (2) e fornire una stima indiretta della PVC. In condizioni di emergenza è utile per indirizzare la diagnosi e la prognosi di patologie cardiache e respiratorie (3) e guidare la somministrazione dei fluidi. Nonostante le notevoli potenzialità, la valutazione del PVG è ormai caduta in disuso. La ragione è sostanzialmente dovuta alla mancanza di una tecnica valutativa che sia al contempo semplice, rapida, non invasiva e il più possibile precisa. La modalità tradizionale di valutazione del PVG si basa su un esame ispettivo semeiologico che visualizza le modifiche del volume della vena giugulare interna mentre il capo è variabilmente inclinato e considerando l’angolo sternale come punto di riferimento per determinare una stima della PVC (4). Nonostante non invasiva, tale valutazione viene considerata di non facile esecuzione e interpretazione (5) e poco accurata. Nella fattispecie è riportato in letteratura che, per quel che riguarda l’accuratezza della stima indiretta della PVC, essa non sia superiore al 50-60% (6). D’altra parte, la misurazione della reale PVC può avvenire solo mediante incannulamento del sistema venoso, approccio pertanto specialistico e poco idoneo alla pratica clinica di routine. A oggi sono stati proposti molti metodi di misurazione non invasiva o minimamente invasiva del PVG e della PVC (6-9), ma nessuna ha dimostrato sufficiente accuratezza e precisione (6) né capacità di fornire informazioni complete al clinico o facilità di uso tali da poter entrare nella normale pratica clinica. Abbiamo pertanto ipotizzato di poter derivare l’onda del PVG tramite una valutazione sequenziale ultrasonografica B-mode non invasiva e in tempo reale delle variazioni dell’area di sezione trasversa della vena giugulare interna durante un ciclo cardiaco. E’ stato già condotto uno studio preliminare pre-clinico in collaborazione con il Dipartimento di Fisica dell’Università di Ferrara, attraverso il quale si è verificato la fattibilità tecnica di questa valutazione dimostrandone l’accuratezza (10). La metodologia messa a punto permetterebbe inoltre, attraverso un algoritmo e un software applicativo, di poter derivare e analizzare l’onda in *post-processing*, già al letto del paziente, minimizzando i tempi di rilevazione e le possibili difficoltà tecniche richiesti all’operatore. Il presente studio si propone di validare questa nuova metodica e di valutarne la trasferibilità in un contesto clinico.

**OBIETTIVI DELLO STUDIO**

**Obiettivo 1:** Validare in un contesto clinico un’innovativa metodica non invasiva basata su una valutazione ultrasonografica B-mode in tempo reale per valutare il PVG (analisi *post-processing* al letto del paziente).

**Obiettivo 2:** Verificare se la metodica proposta può essere trasferita da laboratori di emodinamica e fluidodinamica in un reale contesto clinico, utilizzando le tipologie di apparecchiature ultrasonografiche già largamente presenti negli Ospedali del nostro Paese ed eseguita e interpretata, dopo una preparazione mirata, anche da operatori che non possiedono una specifica qualifica nell’utilizzo della tecnica ultrasonografica.

**DISEGNO SPERIMENTALE OBIETTIVO 1**

Lo sviluppo dell’obiettivo 1 prevede due fasi.

**Fase 1: perfezionamento del software di supporto alla nuova metodica ultrasonografica**

Nello studio preliminare (10) sono state studiate le variazioni dell'area di sezione trasversa del PVG durante un ciclo cardiaco mediante immagini sequenziali ultrasonografiche B-mode in tempo reale ed è stata delineata la forma d'onda risultante. Si è proceduto quindi a verificare se un processo automatico potesse sostituire in modo affidabile la traccia manuale dell’operatore e il risultato preliminare è stato che, grazie a un algoritmo e allo sviluppo di un software originale, la forma d'onda può essere effettivamente tracciata in automatico e analizzata in *post-processing*.

Sulla base dei suddetti dati preliminari, questa prima fase del progetto sarà pertanto dedicata ai seguenti scopi:

a) Al perfezionamento dell'algoritmo e al miglioramento dell’usabilità del software.

b) Allo studio della relazione tra le variazioni di area di sezione trasversa della vena giugulare interna e le variazioni di pressione interne alla vena stessa al fine di sviluppare un modello fisico in grado di fornire una stima indiretta della PVC come misura addizionale a completamento del software.

c) Allo studio delle diverse forme d'onda derivanti dallo studio ultrasonografico della vena giugulare interna in modo da definire quelle normali e quelle caratteristiche di determinate patologie.

Tutto ciò dovrebbe condurre alla messa a punto di un algoritmo e software completi in vista della Fase 2 del progetto che prevede la piena validazione della nuova metodica.

La Fase 1 sarà svolta in stretta collaborazione tra fisici e medici.

Setting

UO di Chirurgia Traslazionale e UO di Anestesiologia e Rianimazione Universitaria dell'Azienda Ospedaliero Universitaria di Ferrara oltre al laboratorio di Fluidodinamica del Dipartimento di Fisica, Università di Ferrara.

Soggetti

-Almeno n = 80 soggetti consecutivi, adulti (> 18 anni) sani di ambo i sessi da sottoporre a valutazione ultrasonografica della vena giugulare interna. Questo numero di soggetti consentirà con ogni probabilità di rilevare e di valutare i diversi sottotipi morfologici della vena giugulare interna.

Criteri di inclusione: soggetti adulti (> 18 anni)

Criteri di esclusione: patologie acute in atto, patologie croniche cardiache o respiratorie, malattie neurodegenerative, gravidanza.

-Almeno n = 50 pazienti consecutivi, adulti, in respiro spontaneo, in regime di ricovero, che necessitano della misurazione della PVC diretta secondo procedura standard, programmata o in emergenza differita. In particolare, una parte di questo campione (n = 25) includerà pazienti affetti da varie patologie cardiache (ad esempio, insufficienza cardiaca congestizia, difetto del setto atriale, insufficienza tricuspidale, fibrillazione atriale, stenosi tricuspidale, blocchi atrio-ventricolari, pericardite costrittiva) al fine di delineare le diverse forme d'onda derivanti con potenziale potere diagnostico.

Criteri di inclusione: ospedalizzati di età > 18 anni in respiro spontaneo che necessitano della misurazione diretta della PVC programmata o in emergenza differita. Parte di questo campione deve presentare patologie cardiache (insufficienza cardiaca congestizia, difetto del setto atriale, insufficienza tricuspidale, fibrillazione atriale, stenosi tricuspidale, blocchi atrio-ventricolari, pericardite costrittiva).

Criteri di esclusione: nessun criterio di carattere clinico. Gravidanza.

La dimensione dei campioni è stata determinata considerando i) i potenziali soggetti sani reclutabili, ii) il numero di pazienti che mediamente afferiscono al reparto di terapia intensiva, iii) il carico di lavoro realmente sostenibile dagli sperimentatori e iv) l'obiettivo della ricerca (ad esempio, n = 50 misure consentiranno di ottenere una forma d'onda tracciata automaticamente dal software derivata da circa 6000 tracciate manualmente).

Metodi

Modalità di acquisizione dell’immagine ultrasonografica: tutti i soggetti, in posizione supina con il collo fermo sull'asse longitudinale e inclinato di 45° all'indietro, verranno sottoposti a scansione ultrasonografica (immagini sequenziali B-mode) della vena giugulare interna destra e sinistra utilizzando una sonda lineare 7,5-11 MHz. Dopo l'acquisizione dell’immagine ritenuta corretta, per ciascun lato del collo verrà effettuata una registrazione di 30 secondi. Le valutazioni saranno eseguite da un operatore esperto e, nel caso di un soggetto in regime di ricovero, in cieco rispetto alle sue condizioni cliniche e alla successiva misura invasiva di PVC. Il tempo complessivo stimato per tale valutazione è di 5 minuti. Nei pazienti ospedalizzati verrà di seguito effettuata la misura diretta di PVC secondo procedura standard dal personale competente.

**Fase 2: processo di validazione della metodica ultrasonografica derivata dalla Fase 1**

Il processo di validazione riguarderà la valutazione del potere diagnostico delle forme di onda rilevate dalla nuova metodica e del valore stimato di PVC con analisi della sensibilità, specificità, valori predittivi positivi e negativi, accuratezza e precisione, ripetibilità.

Setting

UO di Chirurgia Traslazionale e UO di Anestesiologia e Rianimazione Universitaria dell'Azienda Ospedaliero-Universitaria di Ferrara oltre al laboratorio di fluidodinamica del Dipartimento di Fisica dell’Università di Ferrara.

Soggetti

n = 150 pazienti consecutivi, adulti, in respiro spontaneo, in regime di ricovero, che necessitano della misurazione della PVC diretta secondo procedura standard, programmata o in emergenza differita.

Criteri di inclusione: pazienti adulti (> 18 anni) ospedalizzati in respiro spontaneo che necessitano della misurazione diretta della PVC programmata o in emergenza differita.

Criteri di esclusione: nessun criterio di carattere clinico. Gravidanza.

Metodi

Per la verifica della ripetibilità inter-osservatore tutti i pazienti ospedalizzati saranno sottoposti alla valutazione ecografica al proprio letto con le modalità sopra descritte per la Fase 1, in modo indipendente e in sequenza, da due operatori qualificati. Per la verifica della ripetibilità intra-osservatore, gli operatori dovranno ripetere la procedura due volte ed effettuare di ognuna una registrazione di 30 secondi. La valutazione ecografica precederà la misura diretta di PVC già programmata per il paziente nel corso del suo ricovero. Gli operatori saranno in cieco rispetto alle rispettive valutazioni, alla condizione clinica del paziente e alla successiva misura diretta di PVC che verrà eseguita dal personale competente secondo procedura standard.

Per tutte le fasi dello studio la misurazione della PVC non sarà eseguita a finalità sperimentale ma rientrerà nella programmazione prevista per quel paziente nel corso del ricovero. Solo il dato numerico ottenuto verrà considerato allo scopo del presente studio. Non è pertanto prevista alcuna variazione rispetto al normale percorso diagnostico-terapeutico già definito per il paziente.

**DISEGNO SPERIMENTALE OBIETTIVO 2**

Anche il secondo obiettivo del progetto prevede due fasi.

La trasferibilità della nuova tecnica sarà sperimentata in un contesto clinico di emergenza e in uno specializzato. Saranno coinvolti nel progetto quattro clinici senza una specifica competenza nell’utilizzo della tecnica ultrasonografica. Verrà loro chiesto di utilizzare la nuova metodica di valutazione di PVG/PVC, prima (Fase 3) e dopo una preparazione mirata (Fase 4).

Setting = UO di Chirurgia Traslazionale, UO Pronto Soccorso e Medicina d'Urgenza e UO di Neurochirurgia dell’Azienda Ospedaliero Universitaria di Ferrara.

Soggetti: n = 40 pazienti consecutivi che richiedono una valutazione di PVG/PVC programmata o in emergenza differita.

Criteri di inclusione: pazienti > 18 anni che richiedono una valutazione di PVG/PVC programmata o in emergenza differita.

Criteri di esclusione: nessuno di carattere clinico. Gravidanza.

Metodi

Verranno coinvolti quattro clinici appartenenti al reparto di emergenza/urgenza e ad uno specialistico, senza una specifica competenza nell’utilizzo della tecnica ecografica. I clinici riceveranno inizialmente solo un’informazione di base relativa alla nuova metodica di valutazione di PVG/PVC.

Fase 3: ogni clinico valuterà n=10 soggetti con la nuova metodica (indipendentemente, in tempi diversi, ognuno nel proprio contesto clinico). Le misure (due per ogni parte, per valutarne anche la ripetibilità) verranno paragonate con quelle raccolte subito seguentemente da un operatore formato.

Fase 4: lo stesso esperimento verrà ripetuto dai medesimi clinici dopo una specifica e dettagliata formazione per valutare la differenza in accuratezza e precisione rispetto alla fase 3.

La valutazione ecografica sarà addizionale rispetto al normale percorso diagnostico-terapeutico definito per il paziente che non subirà pertanto alcuna variazione.

**ANALISI STATISTICA**

Obiettivo 1-Fase 1. I dati saranno espressi come media e deviazione standard o mediana e range in accordo con la natura delle variabili.

Obiettivo 1 -Fase 2. Le misure derivate dalla nuova metodica saranno paragonate con quelle derivate dal gold standard di misura invasiva di PVC nei pazienti ricoverati in unità operativa di terapia intensiva con gli operatori in cieco rispetto alle condizioni del paziente a ai valori di PVC misurata direttamente. Il numero di pazienti richiesti per stimare la sensibilità e la specificità in una determinata popolazione con una prevalenza del 50%, assumendo un’estensione clinicamente accettabile dell'intervallo di confidenza al 95% del 10%, e una sensibilità e una specificità entrambe di 0.80, è 123. Prevedendo un 20% di valori persi il totale di pazienti richiesti diviene 150. La correlazione fra le due metodiche sarà analizzata mediante il coefficiente di Pearson. Sarà anche utilizzato il di metodo di Bland-Altman per analizzare l’accordo fra le misure derivanti dalle due metodiche. Il processo di validazione comprenderà anche il potere diagnostico della nuova metodica rispetto le diverse forme d’onda, valutando sensibilità, specificità, valori predittivi positivi e negativi, accuratezza e precisione. Saranno utilizzate le curve ROC per valutare sensibilità e specificità corrispondenti a diversi cut-off. Saranno anche calcolate le ripetibilità intra e inter-osservatori.

Obiettivo 2-Fase 3. La dimensione del campione richiesta per valutare la ripetibilità inter/intra-operatori è basato su Walter et al (12). Assumendo un livello minimo di ripetibilità di 0.60 e una ipotesi alternativa di 0.80, un livello alpha di 0.05 e un poter di 0.80, un totale di 40 pazienti sarà necessario per valutare indipendentemente ogni clinico. Sarà determinato il coefficiente di correlazione intraclasse (ICC).

Obiettivo 2-Fase 4. L’ICC sarà determinato anche dopo il corso specifico. Il metodo di Bland-Altman sarà inoltre utilizzato per misurare il livello di accordo fra gli operatori.

L’analisi statistica sarà condotta dal Dipartimento di Salute Pubblica, Medicina Sperimentale e Forense, Unità di Biostatistica ed Epidemiologia Clinica dell’Università di Pavia.

**ANALISI DEI RISCHI, POSSIBILI PROBLEMI E SOLUZIONI**

Il rischio più probabile relativo all’obiettivo 1 è il fallimento del processo di validazione statistico. Per questa ragione si è deciso di procedere a un’analisi ad interim (mese 12). Se l’analisi non risultasse soddisfacente i fisici dovranno rivedere l’ingegnerizzazione del software e/o la precisione del modello fisico prima di continuare l’esperimento.

**IMPORTANZA E INNOVAZIONE**

E’ noto che valutare clinicamente il PVG, per le sue potenzialità diagnostiche e prognostiche, sarebbe buona prassi. Tuttavia, attualmente, la pratica clinica di carattere semeiologico è caduta in disuso (1-9) poiché richiede abilità ed esperienza ormai poco diffuse fra i medici (4,5). A oggi una metodica alternativa strumentale, non-invasiva, rapida ed a basso costo in grado di valutare in maniera completa, precisa e affidabile il PVG e anche la PVC ancora non è stata messa a punto (6). La metodica proposta nel presente progetto, comprensiva di un software specifico di supporto, diversamente da altre in precedenza sviluppate, anch’esse avvalentisi della tecnica ultrasonografica (6-9), potrebbe non solo essere in grado di fornire un valore di stima di PVC, ma anche forme di onda patognomoniche di malattie cardiache. Questa metodica potrebbe essere utile in molteplici condizioni cliniche multidisciplinari di emergenza/urgenza e di elezione.

**DESCRIZIONE DELLA COMPLEMENTARIETÀ E SINERGIA DEL GRUPPO DI RICERCA**

Il progetto prevede un team multidisciplinare di medici, tecnici, fisici e statistici con un ragguardevole background relativo alle tematiche specifiche trattate nel presente progetto, così come una storia di precedenti collaborazioni. L’obiettivo 1-Fase 1 coinvolgerà ricercatori esperti in emodinamica e ultrasonografia del sistema vascolare, e fisici. Questa fase richiede infatti sia competenze mediche che di fisica applicata all’emodinamica, finalizzate alla messa a punto del definitivo algoritmo alla base del software di supporto alla nuova metodica, che sfrutta gli ultrasuoni per la valutazione del PVG e per la stima della PVC. Le misurazioni dirette invasive della PVC previste nella Fase 2 saranno effettuate in un reparto di terapia intensiva, sotto la supervisione di uno sperimentatore esperto responsabile. L’obiettivo 2, che prevede di verificare la trasferibilità della metodica in un contesto clinico, sarà realizzato in sinergia tra clinici dell’UO di emergenza/urgenza, di un’unità specialistica e ricercatori esperti di emodinamica e ultrasonografia. Tecnici qualificati nell’utilizzo degli ultrasuoni in ambito vascolare saranno sempre presenti a supporto di tutte le fasi sperimentali per l'acquisizione delle misurazioni richieste. Infine un’unità di statistici sarà responsabile della validazione della nuova metodica.

**BIBLIOGRAFIA**

1. Naveen G et al. Jugular Venous Pulse : An Appraisal. JIACM, 2000;1(3):260-9.
2. Chua Chiaco JM et al. The jugular venous pressure revisited. Cleve Clin J Med 2013;80(10):638-44.
3. Drazner MH et al. Prognostic importance of elevated jugular venous pressure and a third heart sound in patients with heart failure. N Engl J Med. 2001;345(8):574-81.
4. Mackenzie J. The study of the pulse, arterial, venous and hepatic, and of the movements of the heart. Edinburgh: Young J. Pentland 1902.
5. Applefeld MM. The Jugular Venous Pressure and Pulse Contour. In Clinical Methods: The History, Physical, and Laboratory Examinations. 3rd edition. Boston: Butterworths; 1990.
6. Ward KR et al. A new noninvasive method to determine central venous pressure. Resuscitation. 2006;70(2):238-46.
7. Keller AS et al. Diagnostic accuracy of a simple ultrasound measurement to estimate central venous pressure in spontaneously breathing, critically ill patients. J Hosp Med 2009;4(6):350-5.
8. Deol GR et al. Ultrasound accurately reflects the jugular venous examination but underestimates central venous pressure. Chest 2011;139(1):95-100.
9. Lipton B. Estimation of central venous pressure by ultrasound of the internal jugular vein. Am J Emerg Med 2000;18(4):432-4.
10. Sisini F et al. A ultrasonographic technique to assess the jugular pulse, 2014 (submitted for peer review).
11. Buderer NMF. Statistical methodology: I. incorporating the prevalence of disease in the sample size calculation for sensitivity and specificity. Acad Emerg Med 1996; 3:895-900.
12. Walter SD, Eliasziw M, Donner A. Sample size and optimal designs for reliability studies Statistics in Medicine 1998, 17, 101-110.

**TIMELINE / MILESTONES**

-Mesi 0-6: messa a punto definitiva della nuova metodica ultrasonografica per la valutazione del PVG (obiettivo 1-Fase 1).

-Mesi 6-18: fase sperimentale di raccolta dati per la successiva validazione statistica della metodica (obiettivo 1-Fase 2).

-Mesi 18-21: analisi dei dati relativi all’obiettivo 1.

-Mesi 21-33: valutazione della trasferibilità della metodica ultrasonografica in un contesto clinico multidisciplinare (Obiettivo 2-Fase 3 e Fase 4).

-Mesi 33-36: analisi dei dati relativi all’obiettivo 2 e diffusione dei risultati scientifici.

*Milestones* 18 mesi

Dopo una prima fase (0-6 mesi) dedicati alla piena messa punto della metodica ultrasonografica per la valutazione del PVG, in particolare per il perfezionamento del software che permette la valutazione automatica del PVG e del modello fisico per la stima della CVP, a 18 mesi ci si prefigge la conclusione della parte sperimentale relativa all’obiettivo 1.

*Milestones* 36 mesi

Nel periodo 18-21 mesi verrà eseguita l'analisi dei dati relativi all’ obiettivo 1.

Nel periodo 18-33 mesi verranno condotte le fasi sperimentali dell'obiettivo 2 del progetto, che consiste nella verifica della trasferibilità della nuova metodica in ambito clinico di emergenza/urgenza e di elezione. L'ultimo periodo (33-36 mesi) sarà dedicato all'analisi dei dati finali e alla diffusione dei risultati scientifici dell’intero studio.

**ATTREZZATURE E RISORSE DISPONIBILI**

Le misurazioni ultrasonografiche saranno effettuate da operatori appositamente formati nel corso di dottorati di ricerca dedicati allo studio di parametri non convenzionali della circolazione cerebrale valutata con metodiche ultrasonografiche innovative. Uno strumento Eco-Color Doppler portatile è disponibile nell’UO del PI del progetto (UO di Chirurgia Traslazionale, Azienda Ospedaliero Universitaria di Ferrara), mentre un altro strumento analogo sarà acquisito in leasing per la parte del progetto che prevede le acquisizioni sui pazienti ospedalizzati. Le misurazioni dirette invasive della PVC saranno eseguite nel corso della normale pratica clinica dal personale addetto sotto la supervisione di uno sperimentatore responsabile. I laboratori di fluidodinamica del Dipartimento di Fisica dell’Università di Ferrara (che è coinvolto nel progetto come parte scientifica di supporto e non come unità proponente) è dotato di tecnologie innovative appositamente sviluppate per lo studio del flusso sanguigno in condizioni fisiologiche e patologiche (ad esempio un circuito idraulico che simula la circolazione del sangue nei vasi del collo) e si avvale di un gruppo di ricercatori qualificati nell'analisi dei dati derivati dallo studio dei flussi mediante tecnica ultrasonografica. L’analisi statistica sarà condotta dal Dipartimento di Salute Pubblica, Medicina Sperimentale e Forense, Unità di Biostatistica e Epidemiologia Clinica dell’Università di Pavia (subcontratto). L’intero gruppo di ricerca vanta di una consolidata storia di collaborazioni precedenti.

**RILEVANZA TRASLAZIONALE E IMPATTO PER IL SISTEMA SANITARIO NAZIONALE**

In caso di risultati positivi, la nuova metodica utrasonografica potrebbe essere proposta nella pratica clinica per la valutazione rapida, non invasiva, e a basso costo del PVG e stima della PVC, attraverso l’utilizzo di apparecchiature ecografiche ampiamente disponibili negli Ospedali del nostro Paese, da parte di clinici adeguatamente formati e con un background multidisciplinare. Questa nuova metodica potrebbe risultare utile in molteplici condizioni multidisciplinari di emergenza/urgenza e di elezione.

**ASPETTI ETICI**

Ad ogni paziente il medico descriverà dettagliatamente lo studio e leggerà il foglio informativo consentendogli di porre delle domande ogni qualvolta una parte dovesse risultargli poco comprensibile. Successivamente gli verrà fornita la Scheda di Consenso che egli è tenuto a leggere prima di sottoscrivere (vedi allegati). I dati dei pazienti saranno trattati nel pieno rispetto della legge sulla tutela della privacy. Saranno quindi registrati, elaborati e archiviati in modo da garantire la sicurezza e la riservatezza degli stessi e quando inclusi in pubblicazioni e/o presentazioni a congressi scientifici saranno sottoposti ad elaborazione statistica e quindi trasformati in dati anonimi.
